# Distinguishing between the exponential and Lindley distributions: An illustration from biological psychology

**DOI:** 10.3758/s13428-026-02942-0

**Published:** 2026-02-13

**Authors:** Shovan Chowdhury, Marco Marozzi, Freddy Hernández-Barajas, Fernando Marmolejo-Ramos

**Affiliations:** 1 Decision Sciences and Operations Management, Indian Institute of Management Kozhikode, Kozhikode, Kerala India; 2 Department of Mathematics and Computer Science, University of Ferrara, Ferrara, Italy; 3 Department of Statistics, National University of Colombia, Medellín, Colombia; 4 College of Education, Psychology, and Social Work, Flinders University, Adelaide, Australia

**Keywords:** Censored data, Exponential distribution, Hazard rate function, Likelihood-ratio test, Lindley distribution, Distributional regression

## Abstract

The exponential distribution has been used for modeling positively skewed data in biological psychology. However, the lesser-known Lindley distribution, although not typically used for this purpose, has a density and cumulative distribution that are very similar to those of the exponential distribution. This similarity suggests that the Lindley distribution could be a strong candidate for modeling such data types. While the probability density and cumulative distribution functions of these two one-parameter distributions can be quite similar, their hazard rate functions differ markedly. Therefore, selecting the most appropriate distribution significantly impacts the interpretation of the hazard rate function. To aid in this selection, we introduce a method that distinguishes between the exponential and Lindley distributions by examining the ratio of their maximized likelihood functions. This method is versatile, as it can also be applied to type I censored data, enhancing its practical appeal. Asymptotic results are analytically derived. We conducted a simulation study to demonstrate the method’s effectiveness, even with small sample sizes. Furthermore, we illustrate the method’s application using a published dataset from biological psychology and provide an implementation as an R function.

**Supplementary Information:**

The online version contains supplementary material available at 10.3758/s13428-026-02942-0.

## Introduction

Research in biological psychology frequently encounters data that are positively skewed, meaning the tail of the data distribution extends further to the right, with a concentration of data points at the lower end of the scale and fewer, more extreme values at the higher end. This characteristic is observed in various types of data, including reaction times, certain physiological measures, and scores on psychological scales assessing symptoms or distress. Several factors contribute to the prevalence of positive skew in biological psychology data. For instance, reaction times, a biomarker measure in biological psychology (Jiménez-Figueroa et al., 2017), are inherently bounded by zero (responses cannot be faster than instantaneous) and often exhibit a pattern where most responses are relatively quick, but a smaller number of trials elicit significantly slower responses due to various cognitive or motor factors. Similarly, physiological measures or symptom scores in clinical populations may show a floor effect, where many individuals have low or zero scores (indicating absence or low levels of a symptom/measure), while a smaller subset exhibits higher scores reflecting greater severity or activity (Tomitaka et al., 2017). Effectively modeling such non-normal and skewed data distributions is central for appropriate statistical analysis and interpretation.

To address the characteristics of such positively skewed data, probability distributions are employed. Evidence suggests that the exponential distribution, or models incorporating it, have been widely utilized in biological psychology and related neuroscience fields. A prominent example is the modeling of reaction time data using the ex-Gaussian distribution. The ex-Gaussian distribution is a convolution of a Gaussian (normal) distribution and an exponential distribution. This composite model effectively captures the typical shape of reaction time distributions, with the exponential component accounting for the positively skewed tail (Marmolejo-Ramos et al., 2023). Beyond reaction times, the exponential distribution has been considered in other biological and psychological contexts where the probability of an event occurring remains constant irrespective of elapsed time, thereby facilitating analyses of reaction time variability in cognitive tasks. Some research indicates that the distributions of psychological distress scores in large populations can exhibit an exponential pattern (Tomitaka et al., 2017). Furthermore, in neuroscience, the timing of neuronal firing intervals is sometimes analyzed using exponential distributions, reflecting the stochastic nature of these events (Roberts et al., 2016). Studies in computational neuroscience also note that brain activities can follow distributions belonging to the exponential family, highlighting the relevance of such distributions in understanding neural processes (Changbo et al., in press).

While the exponential distribution, as discussed, has proven a useful model for certain types of positively skewed data in biological psychology, the task of identifying the most appropriate distribution for a given dataset can be challenging when multiple models offer a seemingly good fit. This challenge is particularly relevant when considering distributions with the same number of parameters, such as different one-parameter distributions like the Lindley one that might both provide a good fit for the response variable in the context of distributional regression modeling (Klein, 2024) (e.g., Generalized Additive Models for Location, Scale, and Shape (GAMLSS) modeling). In such cases, standard model comparison criteria like the Akaike information criterion (AIC) or Bayesian information criterion (BIC) estimates may be too close, and the probability density functions (pdfs) and cumulative distribution functions (cdfs) may show significant overlap, making it problematic to definitively decide which distribution is best. While differences between distributions are usually examined via their pdfs or cdfs, it has recently been suggested that examining the hazard rate function (hrf) of the distributions may help to better discriminate between distributions and provide novel information (Kang, 2017; Loeys et al., 2014; Panis et al., 2020).

This study emphasizes the critical need to select the appropriate distribution for accurate interpretation of hrfs and introduces an innovative method based on the ratio of the likelihoods of different distributions. Our study tackles the central issue of distinguishing between the exponential distribution and the less commonly employed but potentially significant Lindley distribution. The proposed discrimination method demonstrates effective handling of type I censored data, making it very useful in practice, given its prevalence in this context.

**Fig. 1:**
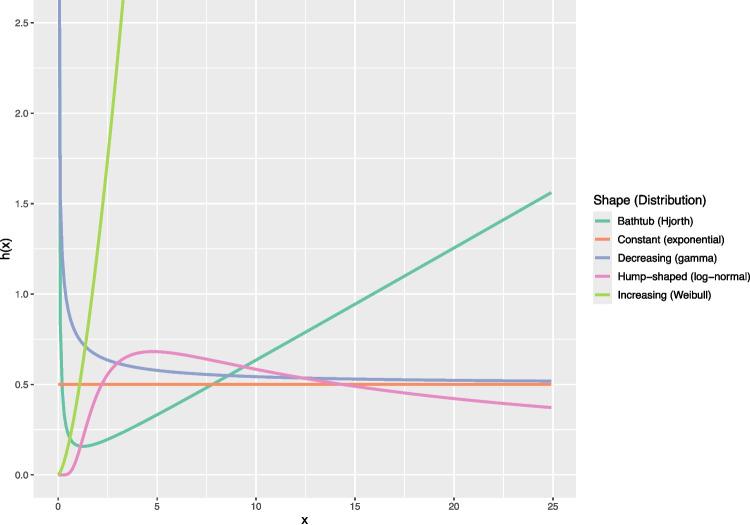
Illustration of hazard rate function *h*(*x*) shapes corresponding to different probability distributions: Hjorth (Bathtub), exponential (Constant), gamma (Decreasing), log-normal (Hump-shaped), and Weibull (Increasing)

The paper is structured as follows. We begin by discussing the importance of selecting an appropriate probability distribution and its influence on interpreting hazard rate functions, followed by an exploration of the role of hazard rate functions in biological psychology data analysis. Next, we examine the statistical properties of the exponential and Lindley distributions. We then introduce our novel discriminating method based on the logarithm of the ratio of maximized likelihoods (RML). Subsequently, we detail the procedure for selecting between the exponential and Lindley distributions using this method, supported by a comprehensive simulation study. We consider the exponential distribution because it is one of the most used distributions in applied psychology. The Lindley distribution is considered because it can be particularly useful in scenarios where the hazard rate might be increasing, offering flexibility. A practical application of the proposed method is demonstrated using data from a biological psychology experiment. Note that scholars in biological psychology typically prefer using distributions like the exponential and Lindley to balance parsimony with empirical usefulness in their quantitative frameworks. Concluding remarks are provided in the final section. Supplementary material, including the Appendix with asymptotic analytical results, implementation of the proposed method in R (R Core Team, 2025) and additional examples and simulation results, is available online in a GitHub repository (see https://github.com/fhernanb/T_exp_lin).

## The influence of probability distributions on hazard rate functions

In linear regression, the location parameter of the dependent variable is modeled by the conditional distribution’s mean, and the normal distribution is used by default. That is, in linear regression, it is assumed that the residuals are normally distributed. In practice, however, continuous dependent variables often deviate from normality, so linear regression may not guarantee normally distributed residuals. Generalized linear models (GLM) extend linear regression by accommodating response variables with various error distribution models from the exponential family, including normal, binomial, Poisson, gamma, and exponential distributions. GAMLSS enhance the capabilities of traditional regression models, such as linear regression and GLMs, by offering several advantages. These include the ability to utilize a broader range of distributions beyond those typically employed in GLMs, thereby providing increased flexibility for different types of data (Klein, 2024; Stasinopoulos et al., 2024). That is, GAMLSS allows the selection of an optimal distribution that best fits the response variable. Furthermore, GAMLSS modeling encourages researchers to explore beyond “mean differences" (Kneib, 2013; Kneib et al., 2023) and to examine all parameters of probability distributions in their statistical analyses (Trafimow et al., 2018).

GAMLSS modeling may reveal scenarios where different distributions with the same number of parameters fit a dataset equally well, as indicated by goodness-of-fit metrics like AIC or BIC. In such cases, the choice of distribution may have minimal practical impact on regression estimates, with the similarity visually confirmed by comparing their pdfs or cdfs (e.g., see Figs. 3 and 4). For example, the probability of a number being smaller than 1 in an exponential distribution with $\lambda =1.944$ and a Lindley distribution with $\theta =2.5$ are 0.8568 and 0.8592 respectively (i.e., $\approx 86\%$); these probabilities are very similar (see the plot in row 1, column 3 in Fig. 4). However, their hrfs convey quite different narratives (see the plot in row 1, column 3 in Fig. 5).

The hrf or failure rate quantifies the instantaneous risk or rate at which an event of interest (such as a response, failure, or occurrence) happens at a specific time *t*, given that the event has not occurred before time *t*. In other words, it represents the likelihood that an event will occur in the next instant, given that it has not occurred up to that point. The hazard rate function *h*(*t*) is defined as the ratio of the probability density function *f*(*t*) to the survival function *S*(*t*). The survival function *S*(*t*) gives the probability that the event has not occurred by time *t*, and it is defined as $S(t)=1-F(t)$, where *F*(*t*) is the cumulative distribution function (i.e., for any given value *x*, the cdf gives the probability that the random variable *X* will take on a value less than or equal to *x*; $F(x) = P(X \le x)$). That is, $h(t)=\frac{f(t)}{S(t)}$. It is thus clear that while the pdf influences the hazard rate function by determining the instantaneous likelihood of the event at a given time (e.g., a higher pdf value at time *t* generally leads to a higher hazard rate function at that time, assuming the survival function does not change drastically), the cdf affects the hazard rate function through the survival function (e.g., as the cdf increases [meaning the probability of the event having occurred by time *t* increases], the survival function decreases. This, in turn, generally leads to an increase in the hazard rate function). All of this underscores that the choice of a probability distribution directly impacts the hazard rate function and its interpretation.

Hazard rate functions can display various shapes, as illustrated in Fig. 1 (also refer to Fig. 1A from Panis et al. (2020)), which consequently affects their interpretation. Some of these shapes include a constant hazard rate function, indicating that the risk of an event remains consistent over time, suggesting a random failure pattern (e.g., exponential and Poisson distributions); an increasing hazard rate function, implying that the risk of an event increases with time, as seen in wear-out processes (e.g., Weibull [with shape parameter > 1] and Lindley distributions); a decreasing hazard rate function, indicating that the risk of an event decreases over time, suggesting improvement (e.g., gamma [with shape parameter < 1] and Pareto distributions); a bathtub-shaped hazard rate function, which combines an initial decreasing hazard rate function, followed by a constant period, and then an increasing hazard rate function (e.g., Hjorth and Chen distributions); and a hump-shaped hazard rate function, characterized by an initial increasing hazard rate function that reaches a peak and then declines (e.g., Birnbaum-Saunders and log-normal distributions). Note also that certain probability distributions can display various hrfs shapes. For instance, when the shape parameters in the two-parameter Weibull and gamma distributions are greater than 1, equal to 1, or less than 1, they exhibit increasing, constant, and decreasing hrfs, respectively.

Examining hrfs offers valuable insights into the changing probability of an event (like a response or an error) over time within biological psychology experiments. Across different tasks that utilize temporal or performance measures, the resulting hazard rate functions can adopt distinct shapes, as the following scenarios illustrate. For instance, consider a task measuring the instantaneous probability of a basic sensory or motor response occurring immediately after a stimulus presentation, where the underlying process is simple and automatic. In such a scenario, involving pure detection or reflexive action without complex decision-making, a constant hazard rate function shape could result, indicating a consistent instantaneous likelihood of response over time once processing begins. A different hrf can occur in a working memory task where participants must retain information in memory, and the risk of error increases as cognitive load accumulates. Such tasks can lead to fatigue or resource depletion effects, and thus an increasing hazard rate function would be expected. A decreasing hazard rate function effectively models the learning curve for a new motor skill, where the high risk of initial errors drops with practice as performance demonstrates adaptation. For complex decision-making, the trajectory of performance–beginning with a high error rate during understanding, stabilizing optimally, and then rising again with fatigue–creates a profile best captured by a bathtub-shaped hazard rate function. Finally, the temporal dynamics of response activation in a priming task, marked by an increasing response probability that peaks and then declines, could be represented by a hazard rate function with a distinct hump shape.

**Fig. 2:**
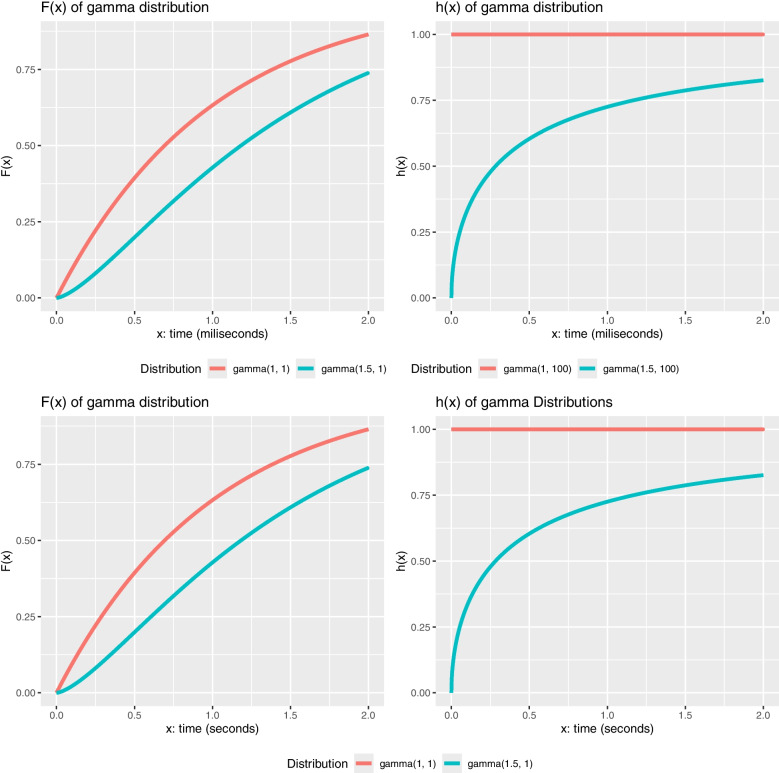
Distributions modeled with gamma distributions. The distributions have positive skews (*first column*) but different hazard rate functions (*second column*). The same data are presented in two analogous metrics

**Fig. 3:**
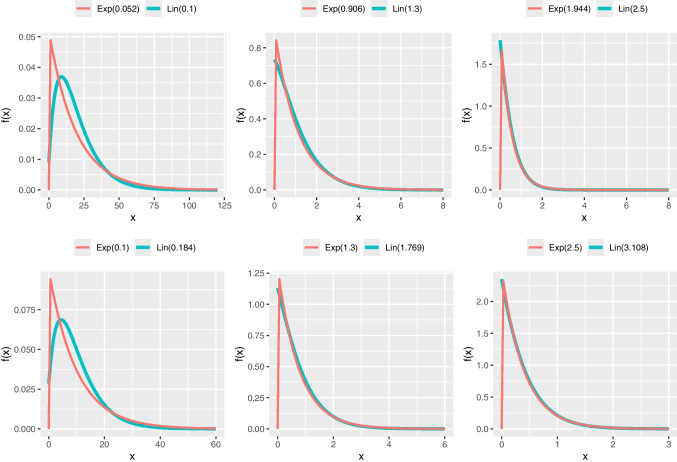
Probability density function *f*(*x*) of Lindley (Lin) and exponential (Exp) distributions for different parameters

**Fig. 4:**
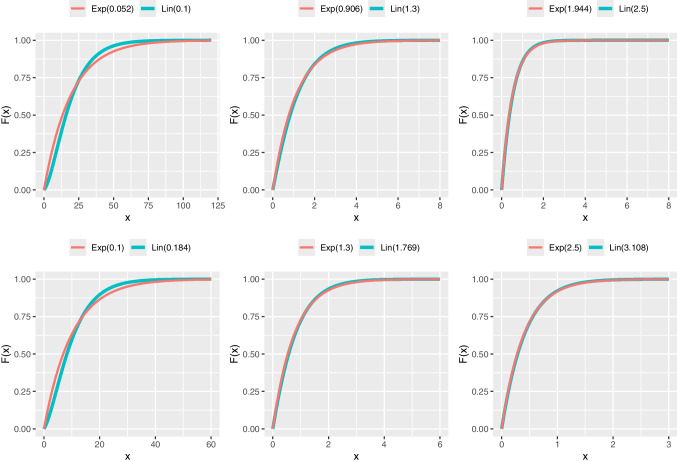
Cumulative density function *F*(*x*) of Lindley (Lin) and exponential (Exp) distributions for different parameters

**Fig. 5:**
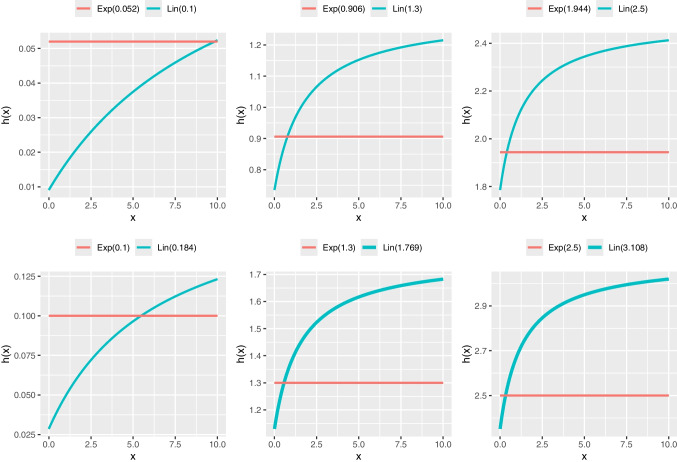
Hazard rate function *h*(*x*) of Lindley (Lin) and exponential (Exp) distributions for different parameters

The units of the hazard rate function are typically expressed as events per unit of time. While the hazard rate function is always non-negative (i.e., there cannot be a negative risk), there is no upper limit on its potential value. To illustrate these concepts with a specific example from cognitive assessment, consider a scenario where participants undergo a digit recognition memory task. In this task, participants are shown a sequence of single digits (0–9) and must quickly identify if a digit has appeared previously in the sequence, under time constraints. One experimental condition, termed ‘immediate recognition’, requires participants to compare the current digit only with the immediately preceding trial. As the working memory load remains constant in this condition, a constant hazard rate function is expected, reflecting the relatively simple comparison process. In contrast, the ‘cumulative recognition’ condition demands that participants compare the current digit against all previously presented digits in the sequence. This leads to an increasing memory load as the sequence progresses. Consequently, an increasing hazard rate function is expected due to the growing demands of memory search. This phenomenon is illustrated in the first row of Fig. 2.

In terms of interpreting the hrf plots, it is evident that the instantaneous rate at which the event occurs differs between the ‘immediate recognition’ and ‘cumulative recognition’ tasks. For instance, at a given time point (e.g., 1000 time units), the hazard rate function is higher in the immediate recognition condition (represented by a gamma(1, 100) distribution with a rate of approximately 0.01 events per time unit) compared to the cumulative recognition condition (represented by a gamma(1.5, 100) distribution with a rate of approximately 0.0096 events per time unit). As illustrated in the plot in the first row, second column of Fig. 2, the probability of the event occurring in the immediate recognition task stays relatively constant over time, assuming the event has not yet occurred. Conversely, the cumulative recognition task shows an increasing probability of the event occurring over time, signaling a growing certainty of a correct identification as the memory search becomes more challenging. The second row in Fig. 2 illustrates this phenomenon using a different time scale (seconds) for the same mock task, further emphasizing the impact of memory load on the hazard rate function.

As the preceding examples illustrate, hrfs offer a powerful means of capturing the temporal dynamics inherent in psychophysiological processes. Because hrfs are fundamentally properties derived from the probability distribution fitted to event-based or time-to-event data, they serve as a versatile tool for investigating temporal structure across various phenomena in biological psychology. This analytical approach is not limited to the specific types of events previously discussed. Many other measures commonly encountered in biological psychology, such as various physiological indices and symptom severities tracked over time, also yield positively skewed data suitable for modeling with similar probability distributions. Consequently, the application and interpretation of hrfs can be broadly extrapolated to these diverse datasets. Analyzing hazard rate functions thus provides a unique, distribution-informed perspective on the temporal unfolding of biological and psychological events, offering insights that go beyond analyses focused merely on central tendency.

## What hazard rate functions offer to data analysis

Panis et al. (2020) argue for using hrfs to model positively skewed data, based on the following points:

- The hrf of response occurrence is much more useful in describing the data than pdf or cdf, being able to emphasize important differences between two situations that are not clear when comparing the corresponding pdfs or cdfs. Two hrfs can be sharply different while the corresponding pdfs or cdfs look very alike (Holden et al., 2009) (see Figs. 3, 4, and 5).
- Townsend (1990) showed that the comparison of hrfs allows stronger conclusions than the comparison of distributions or means, because a complete ordering on the hazard functions implies a complete ordering on the cumulative distribution and survival functions that implies an ordering on the two distribution means, whereas the reverse is not always true. Therefore, an ordering on the two distribution means may happen in the presence of either a complete ordering on the hazard functions or crossing hazard functions. In other words, comparing the means may give a distorted picture of what is really going on.
- Right-censored observations are common when dealing with positively skewed data and can be incorporated into hrf analyses, giving important information often overlooked when using other standard methods (e.g., ANOVA).
- Time-varying explanatory covariates are commonly encountered in biological psychology (e.g., biomarkers related to the respondent autonomic nervous system) and they are easier to incorporate into hrf-based analyses than into standard methods (e.g., ANOVA).
- The hazard function is a critical measure in many biological psychology experiments. It quantifies the instantaneous rate at which an event of interest occurs (e.g., a response or task completion) at a given moment in time, provided the event has not occurred previously. Analyzing hrfs allows for a direct assessment of this instantaneous rate of occurrence over time, whereas standard statistical methods (such as ANOVA) are typically less suited to directly evaluate these specific temporal dynamics.

We thus believe that hrfs are very suitable to examine positively skewed data and in general to compare distributions with different shapes. We thus echo Panis et al. (2020) in that this analytical approach needs to be incorporated into current data modeling practices, particularly in the framework of distributional regression modeling. Figure 5 shows the hazard for various Lindley distributions and shows that it is very different from the hazard for the exponential distribution, which is constant.

## The Lindley and exponential distributions

The Lindley distribution with scale parameter $\theta$, written as Lin$(\theta )$, having probability density function (pdf) and cumulative distribution function (cdf)

4.1 $$\begin{aligned} f_{L}(x;\theta )=\frac{\theta ^2}{1+\theta }\left( 1+x\right) e^{-\theta x};~x,\theta >0, \end{aligned}$$

and

4.2 $$\begin{aligned} F_{L}(x;\theta )=1-\frac{\theta +1+\theta x}{1+\theta } e^{-\theta x};~x,\theta >0, \end{aligned}$$

respectively, was introduced by Lindley (1958). The pdf is decreasing for $\theta \ge 1$ and is unimodal for $\theta <1$. It is also known that the hazard function and the mean residual life (MRL) function of the distribution increase for all $\theta$. Several aspects of this distribution are studied in detail by Ghitany et al. (2008). It has been found that many of the mathematical properties of the Lindley distribution are more flexible than those of the exponential distribution. As pointed out in Ghitany et al. (2008), due to the popularity of the exponential distribution in statistics and many applied areas, the Lindley distribution has not been very well explored in the literature. Still, the Lindley distribution has found its place in many applications where it is found to be preferable over other competitor distributions. For example, this distribution has been used to model lifetime data, especially in applications relating to stress-strength reliability (Lindley, 1958, 1965). Mazucheli and Achcar (2011) used the Lindley distribution to model lifetime data relating to competing risks and Ghitany et al. (2008) used it to model the waiting time of bank customers. That is, the Lindley distribution seems suitable for fitting data akin to lifetime data, i.e., survival times, failure times, or fatigue-life data.

**Table 1:** Key properties of the exponential and Lindley distributions

Distribution	*f*(*x*)	*F*(*x*)	*S*(*x*)	*H*(*x*)	Mean	Median	Variance
exponential	$\lambda e^{-\lambda x}$	$1-e^{-\lambda x}$	$e^{-\lambda x}$	$\lambda$	$\frac{1}{\lambda }$	$\frac{\log 2}{\lambda }$	$\frac{1}{\lambda ^2}$
Lindley	$\frac{\theta ^2}{1+\theta }\left( 1+x\right) e^{-\theta x}$	$1-\frac{\theta +1+\theta x}{\theta +1} e^{-\theta x}$	$\frac{\theta +1+\theta x}{\theta +1} e^{-\theta x}$	$\frac{\theta ^2(1+x)}{\theta +1+\theta x}$	$\frac{\theta +2}{\theta (\theta +1)}$	through iteration	$\frac{\theta ^2+4\theta +2}{\theta ^2(\theta +1)^2}$

*f*(*x*), *F*(*x*), *S*(*x*) and *H*(*x*) represent the pdf, cdf, survival and hazard functions, respectively

The exponential distribution is among the most popular univariate continuous distributions with several significant statistical properties, most importantly, its characterization through lack of memory property. The exponential distribution with scale parameter $\lambda$, written as blue Exp$(\lambda )$, has pdf and cdf given by

4.3 $$\begin{aligned} f_{E}(x;\lambda )=\lambda e^{-\lambda x};~x,\lambda >0 \end{aligned}$$

and

4.4 $$\begin{aligned} F_{E}(x;\lambda )=1-e^{-\lambda x};~x,\lambda >0. \end{aligned}$$

While the pdf of the exponential is decreasing for all $\lambda$, the hazard and MRL functions are constant. This exhibits one of the distinguishable characteristics of exponential and Lindley distributions. However, both one-parameter distributions can be very similarly effective in analyzing positively skewed data. Moreover, the pdfs or cdfs of both distributions can be very close to each other for certain ranges of parameter values. Figures 3 and 4 illustrate the similarity between the distributions when their parameter takes different values. Such similarity translates in that while data could come from the exponential or Lindley distribution, the other distribution could provide a very good fit too.

In Table 1 we present a summary with key properties of the exponential and Lindley distributions. Although the two models may provide a similar fit for small or moderate sample sizes, it is still important to select the best fit for a given data set, and much more importantly to select the correct model in those cases where the difference between a constant and a monotonically increasing hazard function has practical consequences.

The problem of selecting the correct distribution is not new in the statistics literature. The problem of discriminating between two non-nested models for a complete data set was first considered by Cox (1961, 1962) and was later studied by other scholars, including Bain and Engelhardt (1980), and Fearn and Nebenzahl Fearn and Nebenzahl (1991). Due to the increasing applicability of distributions suitable to fit lifetime-like data, special attention has been paid to selecting between the Weibull and log-normal distributions (Kim & Yum, 2008; Kundu & Manglick, 2004), the gamma and log-normal distributions (Kundu & Manglick, 2005), the Lindley and xgamma (Sen et al., 2021), the Lindley, gamma, and Weibull (Raqab et al., 2017), and the exponential and Lindley distributions (Chowdhury, 2019; Vaidyanathan & Varghese, 2019). On the other hand, related literature in the censored data setup is limited and includes papers on selecting between Weibull, log-normal, and gamma distributions for type I censored samples (Quesenberry, 1982), and between Weibull and log-normal distributions for type II censored samples (Cain, 2002; Dey & Kundu, 2012; Kim et al., 2000).

## Discrimination procedure

Data analyzed in biological psychology, as well as in many other fields such as biology and ecology, can be affected by censoring or truncation. While censoring can reduce information by not having access to some observations that are out of range or simply not known, truncation removes existing observations. Censoring can also lead to skewed distributions of the response variable, with an excess of either low (floor effect) or high (ceiling effect) values, and analysis of such data using traditional methods can lead to biased results (Ahmadi et al., 2021; Liu & Wang, 2021).

Two common types of censoring discussed in statistics are type I, where *n* participants are observed up to a fixed time *t*, resulting in a random number of observed events, and type II, where *n* participants are observed until a fixed number of events *r* ($r \le n$) occurs, resulting in a random study duration $t = X_{(r)}$ (the time of the *r*-th event). While these specific censoring schemes are uncommon in typical cross-sectional biological psychology experiments, the underlying issue of incomplete observation can arise. For example, a scenario resembling type I censoring occurs when a researcher imposes a maximum allowable response value; any true response exceeding this limit is only recorded as the maximum, effectively censoring the actual value. An analogue to type II censoring might emerge if a researcher stopped a study once a specific count of participants yielded a response within a defined range, thereby censoring the potential responses of other participants who had not yet met that criterion.

To further clarify how censoring can occur in a biological psychology context, consider a hypothetical longitudinal cognitive neuroscience study. Suppose researchers are following ten patients recovering from traumatic brain injury undergoing a novel cognitive rehabilitation program, periodically assessing their cognitive function using standardized cognitive assessments. The event of interest for analysis could be achieving a specific milestone of cognitive recovery, defined by reaching a pre-defined performance criterion on these assessments. However, not all patients may reach this milestone during the study period, or some might drop out for unrelated reasons, leading to incomplete observation of their event time—a situation known as censoring. Two ways this censoring might be structured, following standard definitions, are:

- *Type I censoring:* The study has a fixed duration, for example, 12 months. For any patient who has not reached the cognitive recovery milestone by the end of 12 months, their event time is censored at 12 months. We know they remained in the ‘at-risk’ state for at least this period.
- *Type II censoring:* The study continues until a fixed number of patients achieve the milestone, for instance, the first seven patients. For the three patients who have not yet reached the milestone when the 7th patient does, their event time is censored. Data collection stops for everyone once the 7th event occurs.

In this section, we describe the selection procedure for the most common right censoring scheme, known as type I censoring. The scheme is briefly described as follows. Let us consider *n* items being observed in a particular experiment. Also suppose that $x_{i:n}, i=1,2,...,n$ be the *i*th ordered observation. In the conventional type I censoring scheme, the experiment is aborted at a pre-determined time $t_0$ such that $x_{t_0}<x_{n:n}$.

Let us first discuss the case of the complete data setting. It is assumed that the data is generated from one of the Exp$(\lambda )$ or Lin$(\theta )$ distributions and the corresponding likelihood functions for the complete data set are respectively

$$\begin{aligned} L_{E}\left( \lambda \right) =\prod _{i=1}^n f_{E}(x_i;\lambda ) \quad \text {and} \quad L_{L}\left( \theta \right) =\prod _{i=1}^n f_{L}(x_i;\theta ), \end{aligned}$$

where $x_i>0$. The ratio of RMLs is defined as $L=\frac{L_{E}\left( \hat{\lambda }\right) }{L_{L}\left( \hat{\theta }\right) };$ when $\hat{\lambda }$ and $\hat{\theta }$ are the maximum likelihood estimators (MLE) of $\lambda$ and $\theta$ respectively. Hence the logarithm of the RML, written as, $T=\log L=l_{E}(\hat{\lambda })-l_{L}(\hat{\theta })$ is obtained as

5.1 $$\begin{aligned} T=n\log \left( \frac{\hat{\lambda }\left( \hat{\theta }+1\right) }{\hat{\theta }^2}\right) +(\hat{\theta }-\hat{\lambda })\sum _{i=1}^n x_i-\sum _{i=1}^n \log (1+x_i). \end{aligned}$$

This step is essential because the log-likelihood ratio statistic is mathematically simpler to compute and analyze compared to the plain likelihood ratio statistic. In the case of the exponential distribution, $\hat{\lambda }$ can be readily derived by differentiating $l_{E}(\lambda )$ with respect to $\lambda$, equating the derivative to zero, and solving for $\lambda$.

5.2 $$\begin{aligned} \hat{\lambda } = \frac{n}{\sum _{i=1}^n x_i}. \end{aligned}$$

Similarly, $\hat{\theta }$, the MLE of the Lindley distribution can be obtained as

5.3 $$\begin{aligned} \hat{\theta }=\frac{-(\bar{x}-1)+\sqrt{(\bar{x}-1)^2+8\bar{x}}}{2\bar{x}}. \end{aligned}$$

The natural model selection criterion will be to choose the exponential distribution, if $T > 0$; otherwise, choose the Lindley distribution (Cox, 1961, 1962). Next, we discuss the selection procedure for the right-censored sample.

**Table 2:** Values for *n* given $\lambda$, *p* and K−S distance between Exp$(\lambda )$ and Lin$(\widetilde{\theta })$ distributions

$\lambda \rightarrow$	0.1	0.5	0.9	1.3	1.5	2.0	2.5
$n~(p=0.6)$	2	8	20	37	49	88	144
$n~(p=0.7)$	9	36	84	159	208	375	617
$n~(p=0.8)$	23	93	216	408	536	967	1590
K-S	0.106	0.054	0.034	0.027	0.021	0.018	0.012

**Table 3:** Values for *n* given $\theta$, *p* and K−S distance between Lin$(\theta )$ and Exp$(\widetilde{\lambda })$ distributions

$\theta \rightarrow$	0.1	0.5	0.9	1.3	1.5	2.0	2.5
$n~(p=0.6)$	1	4	9	18	24	45	78
$n~(p=0.7)$	5	17	40	77	103	194	332
$n~(p=0.8)$	13	45	103	199	265	499	856
K-S	0.120	0.070	0.049	0.036	0.031	0.022	0.015

For type I censoring, the likelihood functions (Lawless, 2003) are given as

$$\begin{aligned} L^*_{E}\left( \lambda \right)&\!=\! \prod _{i=1}^n \left\{ f_{E}(x_i;\lambda )\right\} ^{\delta _i}\left\{ 1 \!-\! F_{E}(t_0;\lambda )\right\} ^{1 \!-\!\delta _i}\quad \text {and}\quad L^*_{L}\left( \theta \right) \\&=\prod _{i=1}^n \left\{ f_{L}(x_i;\theta )\right\} ^{\delta _i}\left\{ 1-F_{L}(t_0; \theta )\right\} ^{1-\delta _i}. \end{aligned}$$

where

5.4 $$\begin{aligned} \delta _i&= 1 \quad \text {if}\quad x_i\le t_0\nonumber \\ \delta _i&= 0 \quad \text {if}\quad x_i>t_0. \end{aligned}$$

The log-likelihood function of $\lambda$ (ignoring constants) is obtained as

$$ l^*_{E}(\lambda )=\log \lambda \sum _{i=1}^n \delta _i-\lambda \sum _{i=1}^n \delta _ix_i-\lambda t_0\sum _{i=1}^n (1-\delta _i). $$

Assuming *d* observations fall below the censoring time $t_0,$ the MLE of $\lambda$ is obtained as

$$\begin{aligned} \hat{\lambda ^*}= &  \frac{\sum _{i=1}^n \delta _i}{\sum _{i=1}^n \delta _ix_i+ t_0\sum _{i=1}^n (1-\delta _i)}\\= &  \frac{d}{\sum _{i=1}^d x_i+ (n-d)t_0}. \end{aligned}$$

Similarly, the log-likelihood function of $\theta$ is obtained as

$$\begin{aligned} l^*_{L}(\theta )= &  2\log \theta \sum _{i=1}^n \delta _i-\theta \sum _{i=1}^n \delta _ix_i+\log (1+\theta +\theta t_0)\sum _{i=1}^n (1-\delta _i)\nonumber \\ &  -n\log (1+\theta )-\theta t_0\sum _{i=1}^n (1-\delta _i)+\sum _{i=1}^n\delta _i \log (1+x_i) \end{aligned}$$.

and the MLE of $\theta$ is obtained iteratively from the following equation

$$\begin{aligned} \frac{2d}{\hat{\theta ^*}}+\frac{(n-d)(1+t_0)}{1+\hat{\theta ^*}+\hat{\theta ^*}t_0}-\frac{n}{1+\hat{\theta ^*}}=\sum _{i=1}^d x_i+(n-d)t_0. \end{aligned}$$

The logarithm of the RML for the right-censored sample in a similar line can be found as

5.5 $$\begin{aligned} T^{*}= &  \sum _{i=1}^n \left[ \delta _i\left\{ \log f_{E}(x_i,\hat{\lambda ^*})-\log f_{L}(x_i,\hat{\theta ^*})\right\} \right. \nonumber \\ &  \left. + (1-\delta _i)\left\{ \log \bar{F}_{E}(t_0,\hat{\lambda ^*})-\log \bar{F}_{L}(t_0,\hat{\theta ^*})\right\} \right] . \end{aligned}$$

The results concerning the asymptotic distribution of *T* and $T^*$, along with the asymptotic mean and variance, are detailed in the Appendix for two different cases, namely when the data come from Exp($\lambda$) and when the data come from a Lin($\theta$).

## Selection procedure and simulation results

In this section, we present the selection procedure (Gupta & Kundu, 2003) for discriminating between the exponential and Lindley distributions. We determine the minimum sample size for a given probability of correct selection (PCS) and tolerance limits, which can be measured by the distance between two cdfs. Practically, the tolerance limit measures the closeness between two cdfs. It is obvious that if the distance between two cdfs is minimal, one needs a very large sample size to discriminate between them for a given PCS. On the other hand, if the cdfs are far apart, a moderate to small sample size may be sufficient to discriminate between the two for a given PCS. Here, we use Kolmogorov–Smirnov (K−S) distance to discriminate between the two cdfs with K−S distance being defined as $\sup _{x} |F(x)-G(x)|$, where *F* and *G* are the cdf of exponential and Lindley distributions, respectively. One may use other distance measures with the same selection criterion. So, the minimum sample size can be determined based on the given PCS (*p*, say) and the tolerance limit (*D*, say) as described in the next subsection.

### Determination of sample size

In view of Theorem 1 in the Appendix, *T* is asymptotically normally distributed with mean $E_E(T)$ and variance $V_E(T)$. The PCS for selecting exponential distribution is given by

$$ PCS(\lambda )=P(T>0~|~\lambda )\approx \Phi \left( \frac{E_E(T)}{\sqrt{V_E(T)}}\right) =\Phi \left( \frac{\sqrt{n}AM_E(T)}{\sqrt{AV_E(T)}}\right) , $$

where $\Phi$ denotes the cdf of the standard normal random variable (*AM* and *AV* stand for asymptotic mean and asymptotic variance respectively). Sample size can be determined by equating the PCS($\lambda$) to the given protection level $p \in [0,1]$ as given by

$$\begin{aligned} \Phi \left( \frac{\sqrt{n}AM_E(T)}{\sqrt{AV_E(T)}}\right) =p \end{aligned}$$

to get

$$\begin{aligned} n=\frac{z^2_{p}AV_E(T)}{\left( AM_E(T)\right) ^2}. \end{aligned}$$

Here, $z_p$ is the p*th* quantile of a standard normal distribution. For different values of $\lambda$, $p=0.6,0.7,0.8$ and K−S distance, sample sizes *n* are reported in Table 2. From this table, it is evident that as $\lambda$ increases, the K−S distance decreases, necessitating a larger sample size. Additionally, it is noticeable that as the probability of correct selection *p* increases, a larger sample size becomes necessary, as anticipated. Proceeding similarly and using Theorem 2 in Appendix A, sample sizes when the true distribution is Lindley, can be determined by the next expression:

$$\begin{aligned} n=\frac{z^2_{p}AV_L(T)}{\left( AM_L(T)\right) ^2}. \end{aligned}$$

Table 3 presents results that exhibit a pattern similar to that observed in Table 2.

Using Theorem 3 in Appendix B, the values of *n* for censored data are obtained. Tables 4 and 5 report the required sample sizes for specified $\lambda$ (or $\theta$), *p* and K−S distance, with 10% censoring. As expected, a higher percentage of censored observations results in larger sample sizes.

**Table 4:** Values for *n* given $\lambda$, *p* and K−S distance between Exp$(\lambda )$ and Lin$(\widetilde{\theta })$ distributions with 10% of censored observations

$\lambda \rightarrow$	0.1	0.5	0.9	1.3	1.5	2.0	2.5
$n~(p=0.6)$	2	11	27	53	72	137	236
$n~(p=0.7)$	10	46	114	229	309	588	1011
$n~(p=0.8)$	26	117	294	590	975	1514	2603
K-S	0.106	0.054	0.034	0.027	0.021	0.018	0.012

**Table 5:** Values for *n* given $\theta$, *p* and K−S distance between Lin$(\theta )$ and Exp$(\widetilde{\lambda })$ distribution with 10% of censored observations

$\theta \rightarrow$	0.1	0.5	0.9	1.3	1.5	2.0	2.5
$n~(p=0.6)$	2	5	13	26	35	70	126
$n~(p=0.7)$	6	22	54	111	152	301	541
$n~(p=0.8)$	15	57	139	285	390	776	1393
K-S	0.120	0.070	0.049	0.036	0.031	0.022	0.015

**Table 6:** PCS based on asymptotic and simulated (in parentheses) results when the data come from Exp$(\lambda )$ distribution for different values of $\lambda$ and *n*

$\lambda \downarrow ~n\rightarrow$	20	40	60	80	100	200
0.1	0.783	0.864	0.912	0.941	0.959	0.993
	(0.712)	(0.840)	(0.899)	(0.936)	(0.958)	(0.995)
0.5	0.652	0.709	0.750	0.782	0.808	0.891
	(0.552)	(0.646)	(0.705)	(0.749)	(0.785)	(0.884)
0.9	0.601	0.641	0.671	0.696	0.716	0.791
	(0.494)	(0.566)	(0.613)	(0.646)	(0.675)	(0.770)
1.3	0.574	0.604	0.626	0.645	0.661	0.722
	(0.451)	(0.517)	(0.562)	(0.589)	(0.611)	(0.692)
1.5	0.567	0.590	0.612	0.634	0.648	0.701
	(0.438)	(0.502)	(0.538)	(0.573)	(0.582)	(0.667)
2.0	0.553	0.575	0.584	0.608	0.617	0.655
	(0.410)	(0.469)	(0.512)	(0.529)	(0.547)	(0.607)
2.5	0.537	0.553	0.564	0.575	0.583	0.617
	(0.403)	(0.454)	(0.484)	(0.500)	(0.523)	(0.584)

**Table 7:** PCS based on asymptotic and simulated (in parentheses) results when the data come from Lin$(\theta )$ distribution for different values of $\theta$ and *n*

$\theta \downarrow ~n\rightarrow$	20	40	60	80	100	200
0.1	0.850	0.930	0.966	0.983	0.988	0.990
	(0.884)	(0.933)	(0.963)	(0.979)	(0.988)	(0.998)
0.5	0.714	0.786	0.832	0.861	0.891	0.965
	(0.780)	(0.828)	(0.861)	(0.889)	(0.908)	(0.963)
0.9	0.644	0.704	0.748	0.773	0.809	0.882
	(0.733)	(0.759)	(0.780)	(0.801)	(0.819)	(0.894)
1.3	0.601	0.656	0.683	0.702	0.727	0.804
	(0.706)	(0.710)	(0.723)	(0.751)	(0.763)	(0.825)
1.5	0.598	0.632	0.655	0.683	0.702	0.776
	(0.698)	(0.699)	(0.707)	(0.722)	(0.731)	(0.791)
2.0	0.577	0.591	0.613	0.634	0.657	0.708
	(0.684)	(0.674)	(0.674)	(0.689)	(0.692)	(0.735)
2.5	0.555	0.579	0.594	0.606	0.615	0.669
	(0.670)	(0.651)	(0.660)	(0.664)	(0.667)	(0.695)

We now discuss the use of PCS and the tolerance level to discriminate between the exponential and Lindley models. Suppose the data are generated from an exponential population, with the tolerance level based on the K−S distance fixed at $D = 0.054$, and the protection level set at $p=0.8$. It implies that discrimination between exponential and Lindley cdfs is desired only when their K−S distance exceeds 0.054. From Table 2, a sample size $n=93$ is required to achieve $p=0.8$ when the data follow an exponential model. Conversely, for data from a Lindley population, Table 3 indicates an approximate $n=103$ for $D=0.054$ and $p=0.8$. Therefore, to meet the protection level $p = 0.8$ at the specified tolerance level, a sample size of $max \{ 93, 103 \} = 103$ is required to satisfy both cases. Although Table 3 does not directly list $D = 0.054$, the adjacent values $D = 0.070$ and $D = 0.049$ suggest that *n* lies close to 103.

For the case of censored data, a similar interpretation follows from Tables 4 and 5. For instance, with $D = 0.054$, $p = 0.8$, and assuming up to 10% censored observations, a sample size of $\max \{ 117, 139 \} = 139$ is required to satisfy both cases simultaneously. The sample sizes in Tables 4 and 5 are slightly higher than those without censoring, as shown in Tables 2 and 3.

**Table 8:** PCS based on asymptotic and simulated (in parentheses) results when the data come from Exp$(\lambda )$ distribution for different values of $\lambda$, *n*, and 10% of censored observations

$\lambda \downarrow ~n\rightarrow$	20	40	60	80	100	200
0.1	0.769	0.850	0.898	0.929	0.950	0.990
	(0.704)	(0.817)	(0.882)	(0.921)	(0.949)	(0.989)
0.5	0.636	0.689	0.726	0.757	0.782	0.864
	(0.530)	(0.629)	(0.669)	(0.715)	(0.747)	(0.844)
0.9	0.587	0.622	0.648	0.670	0.688	0.756
	(0.478)	(0.538)	(0.582)	(0.62)	(0.643)	(0.724)
1.3	0.562	0.587	0.606	0.622	0.635	0.688
	(0.440)	(0.506)	(0.533)	(0.568)	(0.582)	(0.640)
1.5	0.553	0.575	0.591	0.605	0.617	0.664
	(0.431)	(0.491)	(0.528)	(0.544)	(0.564)	(0.623)
2.0	0.539	0.554	0.567	0.577	0.586	0.620
	(0.409)	(0.47)	(0.497)	(0.512)	(0.529)	(0.578)
2.5	0.529	0.542	0.551	0.559	0.566	0.592
	(0.408)	(0.448)	(0.472)	(0.496)	(0.510)	(0.552)

**Table 9:** PCS based on asymptotic and simulated (in parentheses) results when the data come from Exp$(\lambda )$ distribution for different values of $\lambda$, *n*, and 20% of censored observations

$\lambda \downarrow ~n\rightarrow$	20	40	60	80	100	200
0.1	0.757	0.838	0.886	0.918	0.941	0.986
	(0.660)	(0.791)	(0.857)	(0.900)	(0.922)	(0.984)
0.5	0.625	0.674	0.710	0.739	0.763	0.844
	(0.522)	(0.604)	(0.641)	(0.679)	(0.706)	(0.802)
0.9	0.579	0.610	0.634	0.654	0.671	0.735
	(0.459)	(0.532)	(0.571)	(0.587)	(0.609)	(0.684)
1.3	0.555	0.578	0.595	0.609	0.621	0.669
	(0.442)	(0.483)	(0.525)	(0.541)	(0.564)	(0.622)
1.5	0.547	0.567	0.581	0.594	0.605	0.646
	(0.430)	(0.473)	(0.504)	(0.524)	(0.554)	(0.596)
2.0	0.534	0.548	0.559	0.568	0.576	0.606
	(0.416)	(0.463)	(0.485)	(0.509)	(0.509)	(0.552)
2.5	0.526	0.537	0.545	0.552	0.558	0.581
	(0.411)	(0.453)	(0.471)	(0.488)	(0.501)	(0.531)

### Computation of PCS for finite sample sizes

In this subsection, we demonstrate that the asymptotic results derived in the Appendix work well for finite sample sizes. The PCS is computed using both the asymptotic results and Monte Carlo simulations for sample sizes $n=20, 40, 60, 80, 100$, and 200. We first consider the case of a complete sample. Assuming the null distribution to be exponential and the alternative to be Lindley, the results obtained from the asymptotic theory are presented in Table 6 for various choices of the exponential scale parameter $\lambda =0.1, 0.5, 0.9,1.3, 1.5, 2.0, 2.5$. Similarly, for the same sample sizes and corresponding scale parameter $\theta$ of the Lindley distribution, when the null distribution is Lindley and the alternative is exponential, the results are reported in Table 7.

From Tables 6 and 7, clear patterns emerge. As the sample size increases, the asymptotic PCS also increases, as expected. The PCS further increases as the values of $\lambda$ and $\theta$ decrease. Notably, the asymptotic results perform well even for small sample size (e.g., $n=20$) across all parameter ranges. The simulated PCS values, shown in parentheses, follow the same trend and closely agree with the asymptotic PCS.

**Table 10:** PCS based on asymptotic and simulated (in parentheses) results when the data come from Lin$(\theta )$ distribution for different values of $\theta$, *n* and 10% of censored observations

$\theta \downarrow ~n\rightarrow$	20	40	60	80	100	200
0.1	0.833	0.914	0.953	0.973	0.985	0.999
	(0.869)	(0.918)	(0.951)	(0.974)	(0.983)	(0.998)
0.5	0.691	0.76	0.807	0.841	0.868	0.943
	(0.769)	(0.807)	(0.84)	(0.862)	(0.887)	(0.945)
0.9	0.625	0.674	0.710	0.738	0.762	0.843
	(0.706)	(0.739)	(0.753)	(0.78)	(0.796)	(0.858)
1.3	0.588	0.624	0.650	0.672	0.691	0.759
	(0.689)	(0.689)	(0.706)	(0.714)	(0.728)	(0.779)
1.5	0.576	0.606	0.629	0.648	0.665	0.727
	(0.680)	(0.676)	(0.687)	(0.687)	(0.706)	(0.757)
2.0	0.554	0.576	0.593	0.606	0.619	0.665
	(0.666)	(0.650)	(0.648)	(0.666)	(0.665)	(0.685)
2.5	0.540	0.557	0.569	0.580	0.589	0.625
	(0.651)	(0.638)	(0.630)	(0.639)	(0.636)	(0.653)

**Table 11:** PCS based on asymptotic and simulated (in parentheses) results when the data come from Lin$(\theta )$ distribution for different values of $\theta$, *n* and 20% of censored observations

$\theta \downarrow ~n\rightarrow$	20	40	60	80	100	200
0.1	0.820	0.902	0.943	0.966	0.980	0.998
	(0.855)	(0.913)	(0.945)	(0.965)	(0.975)	(0.997)
0.5	0.677	0.742	0.787	0.821	0.848	0.927
	(0.754)	(0.784)	(0.813)	(0.839)	(0.862)	(0.92)
0.9	0.613	0.658	0.691	0.718	0.740	0.819
	(0.702)	(0.709)	(0.720)	(0.748)	(0.763)	(0.832)
1.3	0.579	0.611	0.635	0.655	0.672	0.735
	(0.669)	(0.664)	(0.677)	(0.688)	(0.698)	(0.749)
1.5	0.567	0.595	0.615	0.632	0.647	0.704
	(0.659)	(0.65)	(0.662)	(0.668)	(0.679)	(0.725)
2.0	0.547	0.567	0.582	0.594	0.605	0.647
	(0.653)	(0.642)	(0.631)	(0.639)	(0.645)	(0.662)
2.5	0.535	0.550	0.561	0.570	0.578	0.610
	(0.634)	(0.613)	(0.611)	(0.630)	(0.618)	(0.638)

We also conducted a secondary analysis of the PCS using random samples with censored observations. Tables 8 and 9 present the PCS for 10% and 20% of censoring, respectively, when the random sample is drawn from an exponential distribution. Similarly, Tables 10 and 11 report the PCS for 10% and 20% censoring when the samples are drawn from a Lindley distribution.

To generate censored random samples, we employed a two-step procedure. First, we identified a threshold value $t_0$ corresponding to a specified quantile such that a fixed proportion (10% or 20%) of the observations exceeded $t_0$. Second, observations greater than $t_0$ were replaced by $t_0$ and assigned the indicator $\delta =0$, while those less than $t_0$ were assigned $\delta =1$.

When comparing Tables 6, 8, and 9 for the exponential case, it is evident that increasing the percentage of censored observations leads to a decrease in both asymptotic and simulated PCS, indicating a consistent downward. A similar pattern is observed in the Lindley case when comparing Tables 7, 10, and 11. In all cases, the simulated PCS values closely align with their asymptotic counterparts.

Moreover, when comparing the PCS for the exponential and Lindley distributions under the same level of censoring (complete, 10%, or 20%), the PCS tends to be higher when the samples are drawn from a Lindley population than from an exponential one.

**Table 12:** Goodness-of-fit metrics of the distributions fitted to two data sets

Data set	Distribution	MLE	Max log-likelihood	AIC	BIC	KS (*p* value)
TVSymp | BL	Exponential	2.251	-36.257	74.513	77.772	0.0543 (0.622)
	Lindley	2.837	-36.640	75.279	78.537	0.0500 (0.724)
TVSymp | n-back	Exponential	2.979	17.560	-33.120	-29.863	0.0449 (0.724)
	Lindley	3.623	17.648	-33.296	-30.038	0.0433 (0.846)

**Table 13:** Results from the data analysis

Data set	Distribution	Censoring	Statistic (T)	AM	AV	PCS
TVSymp | BL	exponential	No	0.38243	0.00120	0.00255	0.629
	Lindley	No	0.38243	-0.00114	0.00216	0.633
	exponential	Case 1	0.56303	0.00303	0.00439	
	Lindley					
	exponential	Case 2	0.52071	0.00280	0.00368	
	Lindley					
TVSymp | n-back	exponential	No	-0.0877	0.00062	0.00130	0.594
	Lindley	No	-0.0877	-0.00059	0.00113	0.596
	exponential	Case 1	0.04301	0.00273	0.00378	
	Lindley					
	exponential	Case 2	-0.047211	0.00216	0.00226	
	Lindley					

## Application to biological psychology

Posada-Quintero et al. (2019) had 16 participants undergoing three cognitive tasks; a psychomotor vigilance task (PVT), an auditory working memory task (n-back), and a visual search task (SS). A condition that did not require any psychomotor activity was used as a baseline task (BL). The researchers measured several biomarkers related to the body’s autonomic nervous system (ANS). The participants’ electrodermal activity related to the ANS’ sympathetic component (EDASymp), time-varying index of sympathetic tone (TVSymp), and low- and high-frequency components of heart rate variability (HRVLF and HRVHF) were measured over 12 trials in each task. We have identified two conditional data sets from Posada-Quintero et al. (2019) suitable for Lindley and exponential fits; TVSymp|BL (i.e., time-varying index of sympathetic tone during the baseline task) and TVSymp|n-back (i.e., time-varying index of sympathetic tone during the auditory working memory task). See Supplementary material for details. Table 12 shows the results of the fits for the data sets. The *p* values of the Kolmogorov–Smirnov test indicate that there is not enough evidence that the distributions underlying the data differ from both the Lindley and exponential distributions. Now, we illustrate the application of our proposed statistical test using complete and censored data sets. Although the original data sets are complete, we intentionally censor them to measure the performance of the test. Each data set of size 16 is censored in two ways.

Case 1. The data is censored at the 15th observation so that the 15th and 16th data become identical.

Case 2. The data is censored at the 14th observation so that the 14th–16th data becomes identical.

It is also interesting to study the asymptotic results for such a small sample size of 16. The results for each data set are described below and are shown in Table 13.

### TVSymp | BL data

When exponential and Lindley distributions are used to fit the complete data, maximized log-likelihood functions are given as $l_{E}(2.251)=-36.257$ and $l_{L}(2.837)=-36.640$ respectively resulting in $T=l_{E}(2.251)-l_{L}(2.837)=0.382>0$ which indicates to choose the exponential model. Let us compare this finding with the asymptotic results, as also shown in Table 13. Assuming the data come from exponential cdf, the asymptotic mean and variance are obtained as $AM_E(2.251)=0.00120$ and $AV_E(2.251)=0.0026$ along with the $PCS=0.629$ yielding an estimated risk around 37% to choose the wrong model. Similarly, assuming that the data are from Lindley cdf, we compute $AM_L(2.837)=-0.0011$ and $AV_L(2.837)= 0.0022,$ with the $PCS=0.633$ to yield an estimated risk nearly 27% to choose the wrong model. Therefore, the PCS is at least $min(0.629,0.633)=0.629$. The PCS attains the maximum when the data are coming from the Lindley distribution and hence we should choose the Lindley distribution to fit the data.

### TVSymp | n-back data

The Lindley and exponential distributions also fit the data well (see Table 12). When exponential and Lindley distributions are used to fit the data, maximized log-likelihood functions are given as $l_{E}(2.979)=17.648$ and $l_{L}(3.623)=17.648$ respectively, resulting in $T=l_{E}(2.979)-l_{L}(3.623)=-329+319=-0.088<0$, which indicates to choose the Lindley model. Now, we compute the PCS based on asymptotic results. Assuming that the data come from exponential cdf, the asymptotic mean and variance are obtained as $AM_E(2.979)=0.00062$ and $AV_E(2.979)=0.00129$ along with the $PCS=0.594$ yielding an estimated risk around 40% to choose the wrong model. Similarly, assuming that the data are from Lindley cdf, we compute $AM_L(3.623)=-0.00059$ and $AV_L(3.623)= 0.0011,$ with the $PCS=0.596$ to yield an estimated risk nearly similar to what was obtained using the exponential cdf. Therefore, in this case, the PCS is at least $min(0.594,0.596)=0.594$. The PCS attains the maximum when the data are coming from a Lindley distribution and hence we should choose Lindley distribution to fit the data.

It is noteworthy that, in both datasets, the Lindley distribution demonstrates the most favorable fit according to our methodology. However, based on the AIC, the exponential distribution emerges as the optimal fit in the first dataset, whereas the Lindley distribution exhibits the best fit in the second dataset. While it may be coincidental that the Lindley distribution provides the best fit for both datasets using our method, this consistency simplifies the interpretation and comparison between the two datasets. In the Posada–Quintero and Bolkhovsky study, Posada-Quintero et al. (2019) data were analyzed via ANOVA, task classification analysis (via *k*-nearest neighbor classifiers, support vector machines, decision trees, and discriminant analysis), and classifier performance (via leave-one-subject-out cross-validation). We believe that Lindley distributional regression modeling via GAMLSS (Klein, 2024) would have been helpful to investigate the potential effects of the explanatory variables on the rate scale parameter. In addition, Lindley time-dependent hrf plots would have been informative for estimating the likelihood of an event increasing over time. Specifically, only Lindley hrf plots could have estimated the likelihood of ANS sympathetic tone increasing over time given a cognitive task. If an exponential distribution were the best fit, there would instead be a constant hazard rate function, indicating that a cognitive task has a monotonic likelihood of affecting ANS sympathetic tone over time. Thus, the data example visited above signals that choosing the correct probability distribution has relevant applied consequences, that is, choosing the Lindley or exponential distributions would lead to radically opposite conclusions in an hrf analysis.

## Conclusion

In this paper, we highlight the role of hazard rate functions in discriminating between distributions and show the practical significance of discriminating between two distributions with very similar shapes like the exponential and Lindley ones, suitable to model positively skewed data. We propose a method to address this problem based on the ratio of the likelihood functions. The method is flexible and can be applied to type I censored data, making it very useful in biological psychology as well as in other fields where censored data are common.

It was mentioned earlier that GAMLSS is a framework promoting careful consideration of probability distributions for data interpretation and prediction. As previously explained, hrfs are directly dependent on the pdf and the cdf through the survival function, and they represent an underutilized yet informative approach to analyzing data (see Panis et al. (2020)). We believe that while GAMLSS offers a powerful tool for selecting optimal distributions, our proposed method provides an additional layer of refinement, particularly when dealing with distributions that have an equal number of parameters and similar pdfs and cdfs. By carefully selecting the most suitable distribution, we can optimize the shape of the hrf and enhance the accuracy of our analysis.

## Supplementary Information

Below is the link to the electronic supplementary material.

Supplementary file 1 (pdf 444 KB)

## Data Availability

All data are available on GitHub.

## Appendix A Asymptotic results

For the Borel measurable function *h*(*X*),  $E_E(h(X))$ and $V_E(h(X))$ will denote the mean and variance of *h*(*X*) under the assumptions that *X* follows exponential distribution. Similarly, we define $E_L(h(X))$ and $V_L(h(X))$ as mean and variance of *h*(*X*) under the assumption that *X* follows Lindley distribution. Moreover, if *g*(*X*) and *h*(*X*) are two Borel measurable functions, we define $Cov_E(g(U),h(U))=E_E(g(U)h(U))-E_E(g(U))E_E(h(U))$, and $Cov_L(g(U), h(U))=E_L(g(U)h(U))-E_L(g(U))E_L(h(U)).$ Almost sure convergence will be denoted by *a*.*s*. throughout the paper. We define the following notation for few integrals used in the next subsections.

$$ \Lambda (i,j,k,l)=\int ^{\infty }_{0}\left( \log (1+x)\right) ^i (1+x)^j e^{-(k\lambda +l\theta ) x}dx. $$

### A: Asymptotic results for complete sample

**A1: Exponential distribution as the null hypothesis**

We begin this section with the following Lemma. The proof of the Lemma follows using similar arguments of White (1982) and hence is omitted.

#### Lemma 1

Suppose the data are from $Exp(\lambda$) distribution. Then, as $n\rightarrow \infty$ we have that

(i) $$\hat{\lambda }\rightarrow \lambda ~a.s.$$
(ii) $$\hat{\theta }\rightarrow \widetilde{\theta }~a.s.,$$ where
  $$\begin{aligned} E_{E}\left[ \log f_{L}(x;\widetilde{\theta })\right] =\max \limits _{\theta }E_{E}\left[ \log f_{L}(x;\theta )\right] , \end{aligned}$$
  where $\widetilde{\theta }$ is the quasi-likelihood estimators of $\theta$
(iii) If $T_{*}=l_{E}(\hat{\lambda })-l_{L}(\widetilde{\theta }),$ then $n^{-1/2}(T-E_{E}T)$ is asymptotically equivalent to $n^{-1/2}(T_{*}-E_{E}T_{*}).$

The following theorem follows from the central limit theorem and Lemma 1 (iii), and hence its proof is omitted.

#### Theorem 1

If the data come from *Exp*($\lambda$) distribution, then *T* is approximately normally distributed with mean $E_E(T)$ and variance $V_E(T).$

Now, we discuss how to obtain $\widetilde{\theta }, E_E(T)$ and $V_E(T)$. Let us define

$$\begin{aligned} \Psi _{E}(\theta )= &  E_{E}\left[ \log f_{L}(x;\theta )\right] \nonumber \\ = &  2\log \theta -\log (1+\theta )+\lambda \Lambda (1,0,1,0)-\frac{\theta }{\lambda }. \end{aligned}$$

Differentiating $\Psi _{E}(\theta )$ with respect to $\theta ~(>0)$, we get $\widetilde{\theta }$ after solving the following quadratic equation

$$\begin{aligned} \theta ^2+(1-\lambda )\theta -2\lambda =0. \end{aligned}$$

We observe that $\lim \limits _{n\rightarrow \infty }\frac{E_E(T)}{n}$ and $\lim \limits _{n\rightarrow \infty }\frac{V_E(T)}{n}$ exist. Next we obtain the asymptotic mean and variance of *T* under *Exp*($\lambda$) distribution which are denoted by $AM_E\approx \frac{E_E(T)}{n}$ and $AV_E\approx \frac{V_E(T)}{n}$ respectively and are derived as follows.

$$\begin{aligned} AM_E= &  E_{E}\left[ \log f_{E}(x;\lambda )-\log f_{L}(x;\widetilde{\theta })\right] \nonumber \\= &  \log \lambda \!-\! 2\log \widetilde{\theta } \!+\! \log (1 \!+\! \widetilde{\theta }) \!+\! \frac{\widetilde{\theta }}{\lambda } \!-\! \lambda \Lambda (1,0,1,0) \!-\! 1, \end{aligned}$$

and

$$\begin{aligned} AV_E= &  V_{E}\left[ \log f_{E}(x;\lambda )-\log f_{L}(x;\widetilde{\theta })\right] \nonumber \\= &  \left( \widetilde{\theta }-\lambda \right) ^2 V_E(X)+V_E\left( \log (1+X)\right) \nonumber \\ &  -2(\widetilde{\theta }-\lambda )Cov_E\left( X,\log (1+X)\right) \nonumber \\= &  \left( \frac{\widetilde{\theta }}{\lambda }-1\right) ^2+\lambda \Lambda (2,0,1,0)-\left( \lambda \Lambda (1,0,1,0)\right) ^2 \nonumber \\ &  - 2(\widetilde{\theta }-\lambda )\left[ \lambda \Lambda (1,1,1,0)-(1+\lambda )\Lambda (1,0,1,0)\right] . \end{aligned}$$

**A2: Lindley distribution as the null hypothesis**

We state the following results along the same lines as Lemma 1 and Theorem 1.

#### Lemma 2

Suppose the data are from $Lin(\theta$) distribution. Then, as $n\rightarrow \infty$ we have that

(i) $$\hat{\theta }\rightarrow \theta ~a.s.$$
(ii) $$\hat{\lambda }\rightarrow \widetilde{\lambda }~a.s.$$ where
  $$\begin{aligned} E_{L}\left[ \log f_{E}(x;\widetilde{\lambda })\right] =\max \limits _{\lambda }E_{L}\left[ \log f_{E}(x;\lambda )\right] , \end{aligned}$$
  where $\widetilde{\lambda }$ is the quasi-likelihood estimators of $\lambda$
(iii) If $$T_{**}=l_{E}(\widetilde{\lambda })-l_{L}(\hat{\theta }),$$ then $$n^{-1/2}(T-E_{L}T)$$ is asymptotically equivalent to $$n^{-1/2}(T_{**}-E_{L}T_{**}).$$

As mentioned earlier, the following theorem follows from the central limit theorem and Lemma 2(iii), and hence its proof is omitted.

#### Theorem 2

If the data come from $Lin(\theta$) distribution, then *T* is approximately normally distributed with mean $E_L(T)$ and variance $V_L(T).$

To obtain $\widetilde{\lambda }, E_L(T)$ and $V_L(T)$. Let us define

$$\begin{aligned} \Psi _{L}(\lambda )= &  E_{L}\left[ \log f_{E}(x;\lambda )\right] \nonumber \\ = &  \log \lambda -\frac{\lambda (\theta +2)}{\theta (\theta +1)}. \end{aligned}$$

Differentiating $\Psi _{L}(\lambda )$ with respect to $\lambda$, we get

$$ \widetilde{\lambda }=\frac{\theta (\theta +1)}{\theta +2}. $$

As before, we obtain the expressions of the asymptotic mean and variance of *T* under *Lin*($\theta$) distribution as

$$\begin{aligned} AM_L= &  E_{L}\left[ \log f_{E}(x;\widetilde{\lambda })-\log f_{L}(x;\theta )\right] \nonumber \\= &  \log \widetilde{\lambda }-(\widetilde{\lambda }-\theta )\frac{\theta +2}{\theta (\theta +1)}\nonumber \\ &  +\log \left( \frac{1}{\theta }+\frac{1}{\theta ^2}\right) -\frac{\theta ^2}{\theta +1}\Lambda (1,1,0,1), \end{aligned}$$

and

$$\begin{aligned} AV_L= &  V_{L}\left[ \log f_{E}(x;\widetilde{\lambda })-\log f_{L}(x;\theta )\right] \nonumber \\= &  \left( \theta -\widetilde{\lambda }\right) ^2 V_L(X)+V_L\left( \log (1+X)\right) \nonumber \\ &  -2(\theta -\widetilde{\lambda })Cov_L\left( X,\log (1+X)\right) \nonumber \\= &  \left( \theta -\widetilde{\lambda }\right) ^2\frac{\theta ^2+4\theta +2}{\theta ^2(\theta +1)^2}+\frac{\theta ^2}{\theta +1}\Lambda (2,1,0,1)\nonumber \\ &  -\left( \frac{\theta ^2}{\theta +1}\Lambda (1,1,0,1)\right) ^2\\ &  -2(\theta \!-\! \widetilde{\lambda })\left( \left( \frac{\theta ^2}{\theta \!+\! 1}\left( \Lambda (1,2,0,1) \!-\! \Lambda (1,1,0,1)\right) \right) \right. \nonumber \\ &  \left. -\frac{\theta (\theta +2)}{(\theta +1)^2}\Lambda (1,1,0,1)\right) . \end{aligned}$$

Table 14 shows the estimated values for the asymptotic $AM_E(\lambda )$, $AV_E(\lambda )$ and $\widetilde{\theta }$ for different choices of $\lambda$. From this table we can observe that as $\lambda$ increases, $AM_E(\lambda )$, $AV_E(\lambda )$ go to zero.

**Table 14:** Estimated values of $AM_E(\lambda )$, $AV_E(\lambda )$ and $\widetilde{\theta }$ for different choices of $\lambda$

$\lambda$	$AM_E(\lambda )$	$AV_E(\lambda )$	$\widetilde{\theta }$
0.1	0.0773	0.1952	0.184
0.5	0.0175	0.0401	0.781
0.9	0.0073	0.0162	1.292
1.3	0.0038	0.0083	1.769
1.5	0.0028	0.0062	2.000
2.0	0.0016	0.0033	2.561
2.5	0.0009	0.0020	3.108

Table 15 shows the estimated values for the asymptotic $AM_L(\theta )$, $AV_L(\theta )$ and $\widetilde{\lambda }$ for different choices of $\theta$. From this table, we can observe that as $\theta$ increases, $AM_L(\theta )$, $AV_L(\theta )$ go to zero.

**Table 15:** Estimated values of $AM_L(\theta )$, $AV_L(\theta )$ and $\widetilde{\lambda }$ for different choices of $\theta$

$\theta$	$AM_L(\theta )$	$AV_L(\theta )$	$\widetilde{\lambda }$
0.1	-0.0802	0.1203	0.052
0.5	-0.0275	0.0479	0.300
0.9	-0.0124	0.0223	0.589
1.3	-0.0066	0.0121	0.906
1.5	-0.0049	0.0092	1.071
2.0	-0.0026	0.0049	1.500
2.5	-0.0016	0.0029	1.944

## Appendix B Asymptotic results for censored sample

The asymptotic results for the censored data setting can be easily obtained from the corresponding results for the complete data setting in the previous section.

### Theorem 3

If the data come from *Exp*($\lambda )~(Lin(\theta$)) distribution, then $T^*$ is approximately normally distributed with mean $E_E(T^*)~(E_L(T^*))$ and variance $V_E(T^*)~(V_L(T^*))$, where the asymptotic means and variances of $T^{*}$ assuming exponential and Lindley as null distributions can be given by

$$\begin{aligned} AM_{E}= &  \lim \limits _{n\rightarrow \infty }\frac{E_E(T^*)}{n} = E_{E}\left[ \delta _i\left\{ \log f_{E}(x_i;\lambda )-\log f_{L}(x_i;\theta )\right\} \right. \\ &  \left. +(1-\delta _i)\left\{ \log \bar{F}_{E}(t_0;\lambda )-\log \bar{F}_{L}(t_0;\theta )\right\} \right] , \end{aligned}$$

$$\begin{aligned} AV_{E}= &  \lim \limits _{n\rightarrow \infty }\frac{V_E(T^*)}{n}= V_{E}\left[ \delta _i\left\{ \log f_{E}(x_i;\lambda )-\log f_{L}(x_i;\theta )\right\} \right. \\ &  \left. +(1-\delta _i)\left\{ \log \bar{F}_{E}(t_0;\lambda )-\log \bar{F}_{L}(t_0;\theta )\right\} \right] , \end{aligned}$$

$$\begin{aligned} AM_{L}= &  \lim \limits _{n\rightarrow \infty }\frac{E_L(T^*)}{n}= E_{L}\left[ \delta _i\left\{ \log f_{E}(x_i;\lambda )-\log f_{L}(x_i;\theta )\right\} \right. \\ &  \left. +(1-\delta _i)\left\{ \log \bar{F}_{E}(t_0;\lambda )-\log \bar{F}_{L}(t_0;\theta )\right\} \right] , \end{aligned}$$

$$\begin{aligned} AV_{L}= &  \lim \limits _{n\rightarrow \infty }\frac{V_L(T^*)}{n}= V_{L}\left[ \delta _i\left\{ \log f_{E}(x_i;\lambda )-\log f_{L}(x_i;\theta )\right\} \right. \\ &  \left. +(1-\delta _i)\left\{ \log \bar{F}_{E}(t_0;\lambda )-\log \bar{F}_{L}(t_0;\theta )\right\} \right] , \end{aligned}$$

respectively.

Table 16 presents the estimated values for the asymptotic metrics $AM_E(\lambda )$, $AV_E(\lambda )$, and $\widetilde{\theta }$ for various values of $\lambda$. From this table, it can be observed that as $\lambda$ increases, both $AM_E(\lambda )$ and $AV_E(\lambda )$ approach zero. Similarly, Table 17 displays the estimated values for the asymptotic metrics $AM_L(\theta )$, $AV_L(\theta )$, and $\widetilde{\lambda }$ for different choices of $\theta$. It can be noted from this table that as $\theta$ increases, both $AM_L(\theta )$ and $AV_L(\theta )$ also approach zero (Tables 18 and 19).

**Table 16:** Estimated values of $AM_E(\lambda )$, $AV_E(\lambda )$ and $\widetilde{\theta }$ for different choices of $\lambda$ with 10% of censored observations

$\lambda$	$AM_E(\lambda )$	$AV_E(\lambda )$	$\widetilde{\theta }$
0.1	0.0651	0.1570	0.1844
0.5	0.0130	0.0280	0.7808
0.9	0.0050	0.0105	1.2926
1.3	0.0025	0.0051	1.7694
1.5	0.0018	0.0038	2.0000
2.0	0.0010	0.0019	2.5616
2.5	0.0006	0.0011	3.1085

**Table 17:** Estimated values of $AM_L(\theta )$, $AV_L(\theta )$ and $\widetilde{\lambda }$ for different choices of $\theta$ with 10% of censored observations

$\theta$	$AM_L(\theta )$	$AV_L(\theta )$	$\widetilde{\lambda }$
0.1	-0.0710	0.1084	0.0524
0.5	-0.0226	0.0409	0.3000
0.9	-0.0096	0.0181	0.5897
1.3	-0.0048	0.0092	0.9061
1.5	-0.0035	0.0068	1.0714
2.0	-0.0018	0.0035	1.5000
2.5	-0.0010	0.0020	1.9444

**Table 18:** The values of AM, AV, and estimated $\lambda$ (or $\theta$) for different choices of $\lambda$ (or $\theta$) with 10% of the observations being censored

Exponential case with complete data			
$\lambda$	$AM_E(\lambda )$	$AV_E(\lambda )$	$\widetilde{\theta }$
0.1	0.0773	0.1952	0.184
0.5	0.0175	0.0401	0.781
0.9	0.0073	0.0162	1.292
1.3	0.0038	0.0083	1.769
1.5	0.0028	0.0062	2.000
2.0	0.0016	0.0033	2.561
2.5	0.0009	0.0020	3.108
Lindley case with complete data			
$\theta$	$AM_L(\theta )$	$AV_L(\theta )$	$\widetilde{\lambda }$
0.1	-0.0802	0.1203	0.052
0.5	-0.0275	0.0479	0.300
0.9	-0.0124	0.0223	0.589
1.3	-0.0066	0.0121	0.906
1.5	-0.0049	0.0092	1.071
2.0	-0.0026	0.0049	1.500
2.5	-0.0016	0.0029	1.944
Exponential case with 10% of type I censored data			
$\lambda$	$AM_E(\lambda )$	$AV_E(\lambda )$	$\widetilde{\theta }$
0.1	0.0651	0.1570	0.1844
0.5	0.0130	0.0280	0.7808
0.9	0.0050	0.0105	1.2926
1.3	0.0025	0.0051	1.7694
1.5	0.0018	0.0038	2.0000
2.0	0.0010	0.0019	2.5616
2.5	0.0006	0.0011	3.1085
Lindley case with 10% of type-I censored data			
$\theta$	$AM_L(\theta )$	$AV_L(\theta )$	$\widetilde{\lambda }$
0.1	-0.0710	0.1084	0.0524
0.5	-0.0226	0.0409	0.3000
0.9	-0.0096	0.0181	0.5897
1.3	-0.0048	0.0092	0.9061
1.5	-0.0035	0.0068	1.0714
2.0	-0.0018	0.0035	1.5000
2.5	-0.0010	0.0020	1.9444

**Table 19:** The values of AM, AV, and estimated $\lambda$ (or $\theta$) for different choices of $\lambda$ (or $\theta$) with 10% of the observations being censored

Exponential case with complete data				Exponential case with 10% of type-I censored data			
$\lambda$	$AM_E(\lambda )$	$AV_E(\lambda )$	$\widetilde{\theta }$	$\lambda$	$AM_E(\lambda )$	$AV_E(\lambda )$	$\widetilde{\theta }$
0.1	0.0773	0.1952	0.184	0.1	0.0651	0.1570	0.1844
0.5	0.0175	0.0401	0.781	0.5	0.0130	0.0280	0.7808
0.9	0.0073	0.0162	1.292	0.9	0.0050	0.0105	1.2926
1.3	0.0038	0.0083	1.769	1.3	0.0025	0.0051	1.7694
1.5	0.0028	0.0062	2.000	1.5	0.0018	0.0038	2.0000
2.0	0.0016	0.0033	2.561	2.0	0.0010	0.0019	2.5616
2.5	0.0009	0.0020	3.108	2.5	0.0006	0.0011	3.1085
Lindley case with complete data				Lindley case with 10% of type-I censored data			
$\lambda$	$AM_E(\lambda )$	$AV_E(\lambda )$	$\widetilde{\theta }$	$\lambda$	$AM_E(\lambda )$	$AV_E(\lambda )$	$\widetilde{\theta }$
0.1	-0.0802	0.1203	0.052	0.1	-0.0710	0.1084	0.0524
0.5	-0.0275	0.0479	0.300	0.5	-0.0226	0.0409	0.3000
0.9	-0.0124	0.0223	0.589	0.9	-0.0096	0.0181	0.5897
1.3	-0.0066	0.0121	0.906	1.3	-0.0048	0.0092	0.9061
1.5	-0.0049	0.0092	1.071	1.5	-0.0035	0.0068	1.0714
2.0	-0.0026	0.0049	1.500	2.0	-0.0018	0.0035	1.5000
2.5	-0.0016	0.0029	1.944	2.5	-0.0010	0.0020	1.9444

## Declarations

### Code availability

All code are available on GitHub: https://github.com/fhernanb/T_exp_lin.
