# Supplementary material for: Distinguishing between the exponential and Lindley distributions: An illustration from biological psychology
Source: Behav Res Methods. 2026 Feb 13;58(2):63. doi: 10.3758/s13428-026-02942-0 (PMC12904947; doi:10.3758/s13428-026-02942-0)
Supplement: Supplementary file 1 — (pdf 444 KB) [file 13428_2026_2942_MOESM1_ESM.pdf]

Supplementary material for the paper "Distinguishing between the exponential and Lindley distributions. An Illustration from Biological Psychology"

by authors

January 7, 2026

1 R function to perform the proposed test

Additionally to the proposed test, we created an R function to allow using the test. The R function is called `exp_lin_test()` and belongs to the general class `htest`. The arguments for the `exp_lin_test()` function are:

- `y`: the vector with the random sample. If the random sample contains censored observations, the vector must be created using the `Surv` function from the `survival` R package. The third and fourth examples demonstrate how to use this function to create an appropriate input vector.
- `alternative`: it corresponds to alternative hypothesis H_A, the two possibilities are `"not.exp"` or `"not.lin"`.
- `type`: a string with the censoring type in the vector `y`. The possible options are `"complete"` or `"I"`.

The proposed function is available in the GitHub repository T_exp_lin available in `https://github.com/fhernanb/T_exp_lin`. Below there are some examples using the function `exp_lin_test()`. Before using the proposed function, we must load the main and auxiliary functions from the repository using the next code.

```
url <- "https://raw.githubusercontent.com/fhernanb/T_exp_lin/main/Scripts/exp_lin_test.R"
source(url)

library(RelDists)     # R package with reliability dists. To use dLIN distribution
library(survival)     # To manage censored data
```

1.1 First example

In the first example, we simulate 500 observations from the $Exp(\lambda = 3)$, and the null hypothesis in this example is H_0 : the random sample comes from an exponential distribution. Then we apply the `exp_lin_test()` function with the argument `alternative="not.exp"`. Below is the code for this example.

```r
# Exponential case
lambda <- 3
nobs <- 500
set.seed(123456)
y <- rexp(n=nobs, rate=lambda)

# Applying the test
exp_lin_test(y, alternative="not.exp", type="complete")
```

```
        Exponential-Lindley test

data:
T = 0.062172, p-value = 0.9433
alternative hypothesis: not.exp
sample estimates:
  lambda_hat     AM for T     AV for T
2.8045187901 0.0007161487 0.0015070200
```

The `exp_lin_test()` function gives a list with the T-value, either $\hat{\lambda}$ or $\tilde{\theta}$ (according to the selected null hypothesis), the average mean (AM) and average variance (AV) of the normal distribution for the statistic T and the p-value. From the last results, we observe that $T = 0.0622 > 0$ and p-value is 0.9433; those values suggest not rejecting the null hypothesis that the random sample comes from an exponential distribution.

1.2 Second example

In the second example, we simulate 500 observations from the $Lin(\theta = 3)$ and the null hypothesis in this example is H_0 : the random sample comes from a Lindley distribution. Then we apply the `exp_lin_test()` function with the argument `alternative="not.lin"`. Below is the code for this example.

```r
# Lindley case
theta <- 3
nobs <- 500
set.seed(123456)
y <- rLIN(n=nobs, mu=theta)

# Applying the test
exp_lin_test(y, alternative="not.lin", type="complete")
```

```
        Exponential-Lindley test

data:
T = -0.076938, p-value = 0.9538
alternative hypothesis: not.lin
sample estimates:
   theta_hat     AM for T     AV for T
 2.904103496 -0.001069383  0.002032305
```

From the last results we observe that $T = -0.0769 < 0$ and p-value is 0.9538, those values suggest to do not reject the null hypothesis that the random sample comes from a Lindley distribution.

1.3 Third example

In this example, we illustrate how to use `exp_lin_test` with censored data type I. We assume that the study is closed at $t_0 = 0.76$, indicating that approximately 10% of the observations are censored. Below is the code to generate a random vector with only 500 observations taken from an exponential distribution with $\lambda = 3$.

```
# Exponential case and type I
lambda <- 3
t0 <- 0.76 # End of the study to create censored observations
nobs <- 500
set.seed(1234567)
y <- rexp(n=nobs, rate=lambda)
delta <- rep(1, times=length(y))
delta[y >= t0] <- 0
y[y >= t0] <- t0
y <- Surv(y, delta, type="right") # Converting y to Surv class

# Applying the test
exp_lin_test(y, alternative="not.exp", type="I")

        Exponential-Lindley test

data:
T = 0.44312, p-value = 1
alternative hypothesis: not.exp
sample estimates:
  lambda_hat     AM for T     AV for T
3.2219872919 0.0005768928 0.0008687534
```

From the last output, we do not reject H_0 due to the p-value. Additionally, we obtain that $\hat{\lambda} = 3.2220$ which is near to the true value of $\lambda = 3$.

1.4 Fourth example

In this example, we illustrate how to use `exp_lin_test` with censored data type I. We assume that the study is closed at $t_0 = 0.94$, indicating that approximately 10% of the observations are censored. Below is the code to generate a random vector with only 500 observations taken from an exponential distribution with $\theta = 3$.

```
# Lindley case and type I
theta <- 3
t0 <- 0.94 # End of the study to create censored observations
nobs <- 500
set.seed(1234567)
```

```r
y <- rLIN(n=nobs, mu=theta)
delta <- rep(1, times=length(y))
delta[y >= t0] <- 0
y[y >= t0] <- t0
y <- Surv(y, delta, type="right") # Converting y to Surv class

# Applying the test
exp_lin_test(y, alternative="not.lin", type="I")

        Exponential-Lindley test

data:
T = -0.4613, p-value = 1
alternative hypothesis: not.lin
sample estimates:
    theta_hat       AM for T       AV for T
 2.9402362246 -0.0007707087  0.0015876969
```

From the last output, we do not reject H_0 due to the p-value. Additionally, we obtain that $\hat{\theta} = 2.9402$ which is near to the true value of $\theta = 3$.

2 Application to Experimental Psychology

In this section we present the procedure to apply the proposed test for the data in Posada-Quintero and Bolkhovsky [1].

First, we load the original dataset and then create two sub-datasets, one for the BL condition and the other for the n-back condition using only information of the initial trial of the expermient.

```r
library(readxl)
library(gamlss)
library(RelDists)
library(dplyr)

url_data <- "https://raw.githubusercontent.com/fhernanb/T_exp_lin/main/data/MultiTask_indice
dataset <- read.table(file=url_data, header=TRUE)

dataset |>
  filter(condition == "BL" & trial == 1) |>
  select(TVSymp) -> data_bl

dataset |>
  filter(condition == "n_back" & trial == 1) |>
  select(TVSymp) -> data_nback
```

To estimate the parameters of exponential and Lindley distributions for the BL condition we can use the gamlss function as follows.

```
# Part 1 for TVSymp | BL data ---------------------------------------------
mod1 <- gamlss(TVSymp ~ 1, family=EXP, data=data_bl)
1/exp(coef(mod1))

mod2 <- gamlss(TVSymp ~ 1, family=LIN, data=data_bl)
exp(coef(mod2))
```

The output of the last code gives that $\hat{\lambda} = 3.108$ and $\hat{\theta} = 3.761$. To apply the proposed test, we can use the next code.

```
url <- "https://raw.githubusercontent.com/fhernanb/T_exp_lin/main/Scripts/exp_lin_test.R"
source(url)
exp_lin_test(data_bl$TVSymp, alternative="not.exp", type="complete")

        Exponential-Lindley test

data:
T = 0.027597, p-value = 0.7859
alternative hypothesis: not.exp
sample estimates:
  lambda_hat     AM for T     AV for T
3.1082996191 0.0005559361 0.0011649571
```

The last result indicates that there is evidence not to reject the null hypothesis of the random sample coming from an exponential distribution.

In the next figure, we have the histogram for the TVSymp under BL condition with the two estimated curves, $Exp(\hat{\lambda} = 3.108)$ and $Lin(\hat{\theta} = 3.761)$.

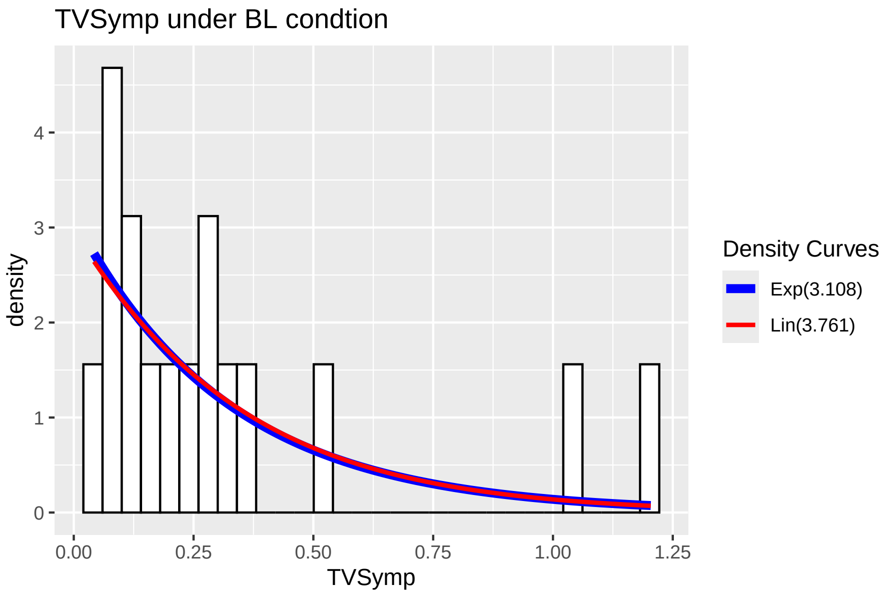

Figure 1: Histogram for TVSymp under BL condition with the two estimated densities functions.

In a similar way, we can estimate the parameters of exponential and Lindley distributions for the n-back condition; we can use the gamlss function as follows.

```
# Part 2 for TVSymp | n back ----------------------------------------------------------
mod3 <- gamlss(TVSymp ~ 1, family=EXP, data=data_nback)
1/exp(coef(mod3))

mod4 <- gamlss(TVSymp ~ 1, family=LIN, data=data_nback)
exp(coef(mod4))
```

The output of the last code gives that $\hat{\lambda} = 3.853$ and $\hat{\theta} = 4.548$. To apply the proposed test we can use the next code.

```
exp_lin_test(data_nback$TVSymp, alternative="not.exp", type="complete")

        Exponential-Lindley test

data:
T = -0.14319, p-value = 1.319e-08
alternative hypothesis: not.exp
sample estimates:
  lambda_hat      AM for T      AV for T
3.8529631388 0.0003200969 0.0006652872
```

The last result indicates that there is evidence to reject the null hypothesis of the random sample comes from an exponential distribution.

In the following figure we have the histogram for the TVSymp under BL condition with the two estimated curves, $Exp(\hat{\lambda} = 3.853)$ and $Lin(\hat{\theta} = 4.548)$.

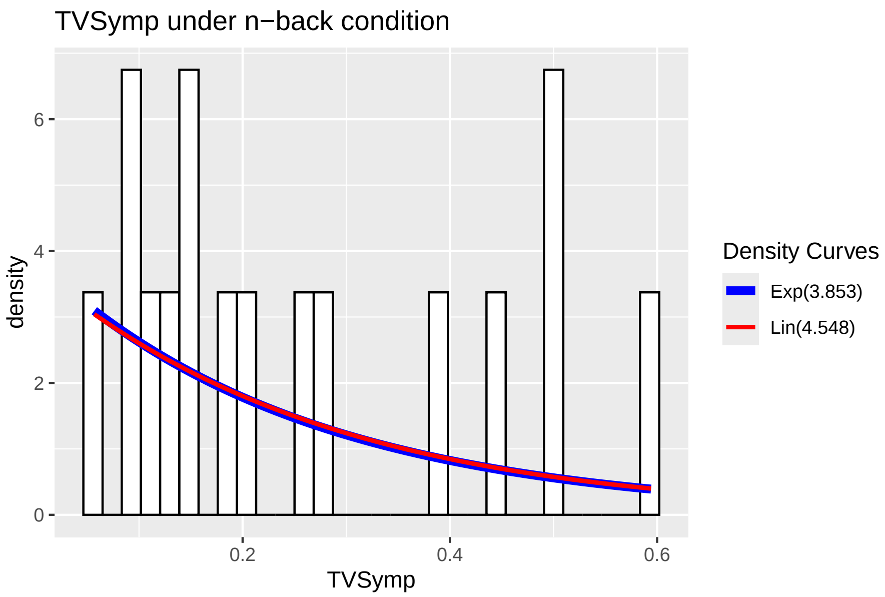

Figure 2: Histogram for TVSymp under BL condition with the two estimated densities functions.

3 Simulation study

In this section, we present graphically the results of a Monte Carlo simulation study to evaluate the performance of the T statistic to identify the best fitting distribution (exponential or Lind-

ley) for simulated data sets. The simulation was performed using R and all codes are available in the next github repository: `https://raw.githubusercontent.com/fhernanb/T_exp_lin/main/Scripts/simul_comp_cens.R`.

Three scenarios were considered in the simulation study:

- random sample with complete data,
- random sample with 10% type I censoring.
- random sample with 20% type I censoring.

We simulate random samples of size n from a population with a general parameter μ (representing θ in expression 1 or λ in expression 2) and a certain of percentage of right censored observations. The censored random samples were generated using the 90th and 80th percentiles, denoted as t_0, for each distribution. Observations greater than t_0 were replaced by t_0, with the indicator $\delta = 0$, while observations less than or equal to t_0 were assigned the indicator $\delta = 1$.

Below there are the expressions for the density function for the Lindley and exponential distributions.

$$f_L(x;\theta) = \frac{\theta^2}{1+\theta}\,(1+x)\,e^{-\theta x};\ x,\theta > 0, \tag{1}$$

and

$$f_E(x;\lambda) = \lambda e^{-\lambda x};\ x,\lambda > 0. \tag{2}$$

Figure 3 depicts the density function across various parameter values. This figure illustrates how the distributions become more similar as the parameter value increases. For this reason, the proposed test is useful for accurately identifying the actual distribution of the random sample.

The levels for the different factors considered in the simulation study are shown below.

- Distribution of the random sample: exponential or Lindley.
- Value for the parameter $\mu = 0.1, 0.5, 0.9, 1.3, 1.5, 2.0, 2.5$.
- Size of the random sample $n = 20, 40, 60, 80, 100, 200$.
- Percentage of censored data for type I: $0\%, 10\%, 20\%$.

The performance of the proposed method is measured with the probability of correct model selection (PCS) to distinguish between exponential and Lindley distributions.

3.1 PCS for a random sample with complete data

Figure 4 displays the results of the probability of correct model selection (PCS) for cases where the true distribution of the random sample follows an exponential distribution (left panel) or a Lindley distribution (right panel).

From this figure, we can observe clear patterns: as the sample size increases, the probability of correct model selection also tends to increase. For lower values of μ, the probability of correct model selection tends to be higher. This could be attributed to the differences in density between both distributions when μ is lower (see Figure 3 panel A). For the case where the true distribution of the random sample is Lindley (right panel), we observe a slightly greater probability of correct model selection compared to the exponential case (left panel).

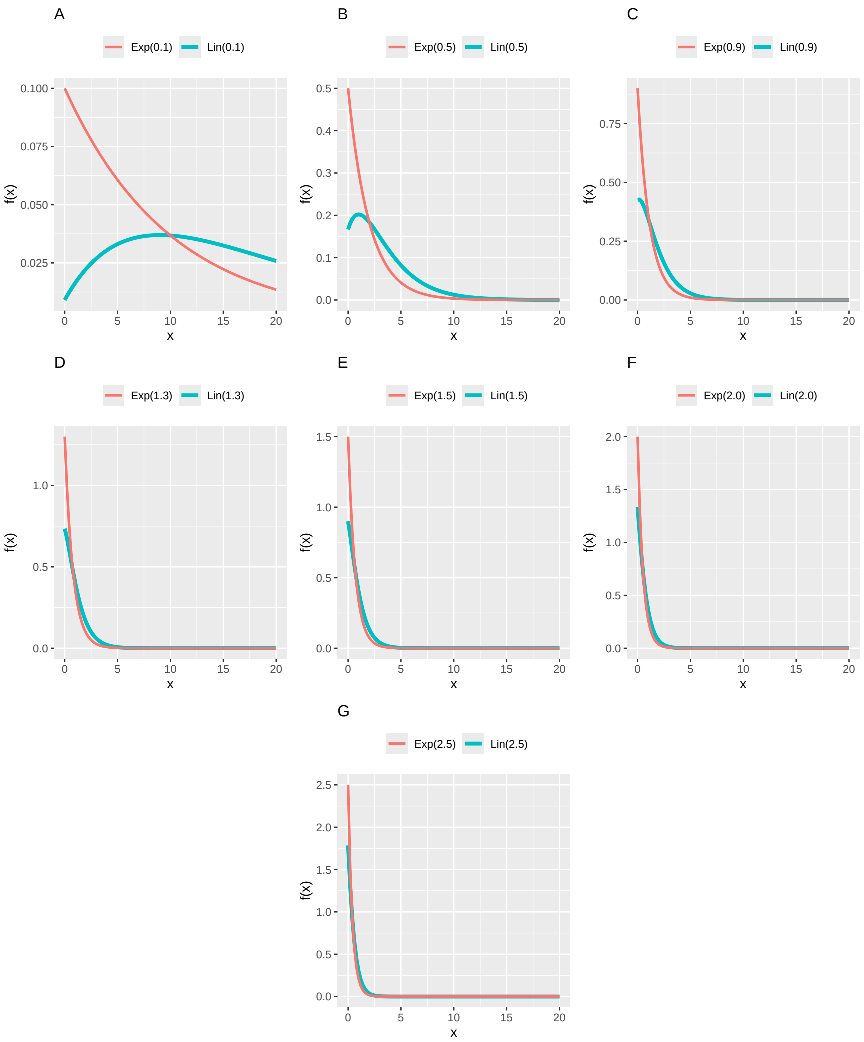

Figure 3: Density function for the Lindley (Lin) and exponential (Exp) distributions for some values of the parameters.

3.2 Results for a random sample with type I censoring

Figures 5 and 6 display the results of the probability of correct model selection (PCS) when the random sample is obtained under a Type I censoring scheme. In these figures, we can observe the same patterns as those found in the case with complete data. Comparing Figures 4, 5, and 6, it is evident that as the percentage of censored observations increases, the PCS tends to decrease. This indicates that the presence of censored observations negatively affects the PCS of the proposed test.

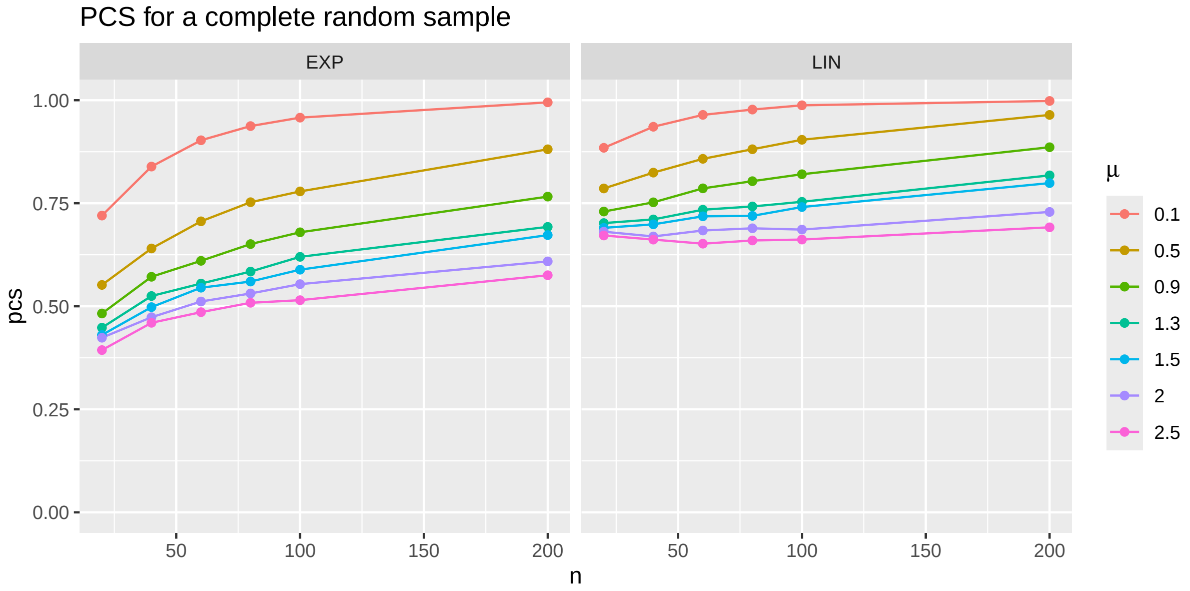

Figure 4: Probability of correct selection (PCS) given the true distribution, sample size n, different values of μ and complete data.

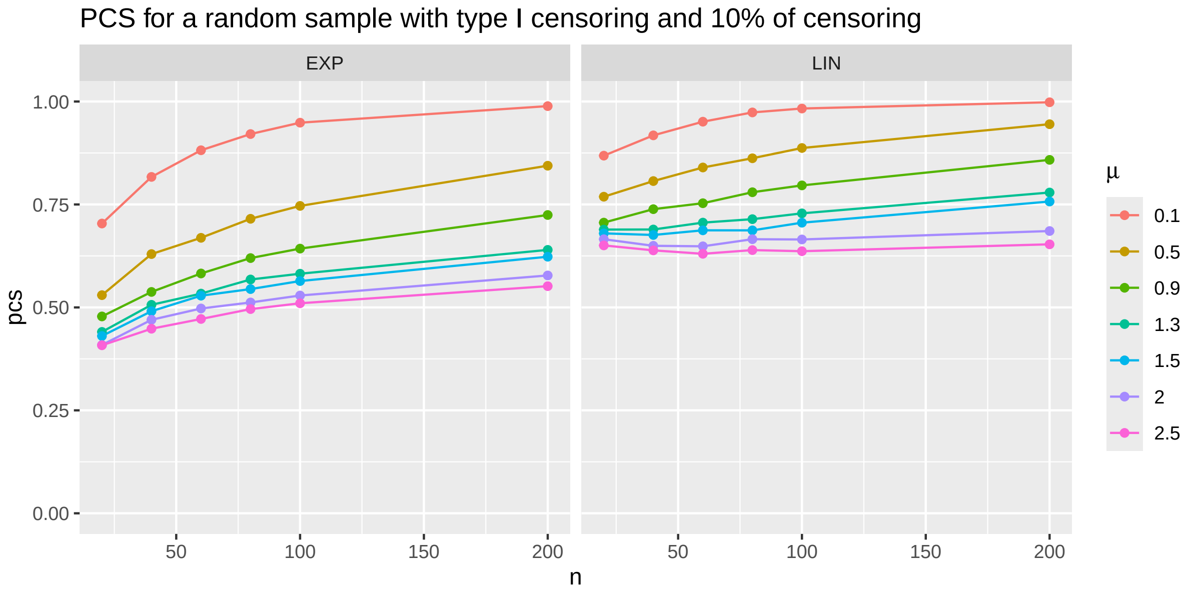

Figure 5: Probability of correct selection (PCS) given the true distribution, sample size n, different values of μ and 10% of type I censoring.

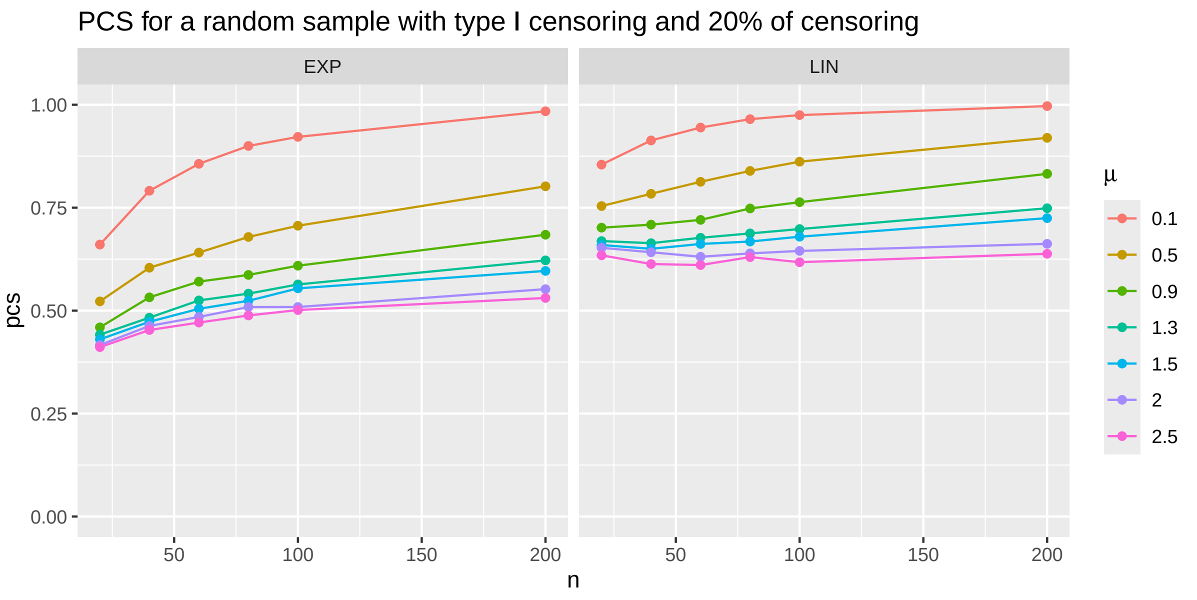

Figure 6: Probability of correct selection (PCS) given the true distribution, sample size n, different values of μ and 20% of type I censoring.

4 R functions to obtain PCS

We also propose four vectorized functions to obtain the PCS in four scenarios: complete data assuming an exponential distribution, complete data assuming a Lindley distribution and censored data assuming an exponential and Lindley distribution. In the following, we summarize the four functions with their parameters.

- `PCS_exp_complete(lambda, n)`.
- `PCS_lin_complete(theta, n)`.
- `PCS_exp_censored(lambda, n, delta)`.
- `PCS_lin_censored(theta, n, delta)`.

Any user could run the following code in the R console to load the functions.

```
# To load the functions
url <- "https://raw.githubusercontent.com/fhernanb/T_exp_lin/main/Scripts/exp_lin_test.R"
source(url)
url <- "https://raw.githubusercontent.com/fhernanb/T_exp_lin/refs/heads/main/Scripts/PCS.R"
source(url)
```

For example, we can use the next code to obtain the PCSs reported in Tables 6 to 11 in the main paper.

```
# Example with two cells from Table 6
PCS_exp_complete(lambda=c(0.1, 0.5), n=c(20, 40))
```

```
[1] 0.7830057 0.7094604
```

```r
# Example with two cells from Table 7
PCS_lin_complete(theta=c(0.1, 0.5), n=c(20, 40))
```

```
[1] 0.8495599 0.7864485
```

```r
# Example with two cells from Table 8
PCS_exp_censored(lambda=c(0.1, 0.5), n=c(20, 40), delta=0.9)
```

```
[1] 0.7686053 0.6885142
```

The last obtained PCS are the same as the PCS reported in Tables 6, 7, and 8; please check Figure 7 with a screenshot of Tables 6, 7, and 8, with red arrows; we highlighted the PCS obtained in the last output.

$\lambda \downarrow$ $n \rightarrow$	20	40	60	80	100	200
0.1	0.783	0.864	0.912	0.941	0.959	0.993
	(0.712)	(0.840)	(0.899)	(0.936)	(0.958)	(0.995)
0.5	0.652	0.709	0.750	0.782	0.808	0.891
	(0.552)	(0.646)	(0.705)	(0.749)	(0.785)	(0.884)
0.9	0.601	0.641	0.671	0.696	0.716	0.791
	(0.494)	(0.566)	(0.613)	(0.646)	(0.675)	(0.770)
1.3	0.574	0.604	0.626	0.645	0.661	0.722
	(0.451)	(0.517)	(0.562)	(0.589)	(0.611)	(0.692)
1.5	0.567	0.590	0.612	0.634	0.648	0.701
	(0.438)	(0.502)	(0.538)	(0.573)	(0.582)	(0.667)
2.0	0.553	0.575	0.584	0.608	0.617	0.655
	(0.410)	(0.469)	(0.512)	(0.529)	(0.547)	(0.607)
2.5	0.537	0.553	0.564	0.575	0.583	0.617
	(0.403)	(0.454)	(0.484)	(0.500)	(0.523)	(0.584)

Table 6: PCS based on asymptotic and simulated (on parenthesis) results when the data come from $Exp(\lambda)$ distribution for different values of λ and n.

$\theta \downarrow$ $n \rightarrow$	20	40	60	80	100	200
0.1	0.850	0.930	0.966	0.983	0.988	0.990
	(0.884)	(0.933)	(0.963)	(0.979)	(0.988)	(0.998)
0.5	0.714	0.786	0.832	0.861	0.891	0.965
	(0.780)	(0.828)	(0.861)	(0.889)	(0.908)	(0.963)
0.9	0.644	0.704	0.748	0.773	0.809	0.882
	(0.733)	(0.759)	(0.780)	(0.801)	(0.819)	(0.894)
1.3	0.601	0.656	0.683	0.702	0.727	0.804
	(0.706)	(0.710)	(0.723)	(0.751)	(0.763)	(0.825)
1.5	0.598	0.632	0.655	0.683	0.702	0.776
	(0.698)	(0.699)	(0.707)	(0.722)	(0.731)	(0.791)
2.0	0.577	0.591	0.613	0.634	0.657	0.708
	(0.684)	(0.674)	(0.674)	(0.689)	(0.692)	(0.735)
2.5	0.555	0.579	0.594	0.606	0.615	0.669
	(0.670)	(0.651)	(0.660)	(0.664)	(0.667)	(0.695)

Table 7: PCS based on asymptotic and simulated (on parenthesis) results when the data come from $Lin(\theta)$ distribution for different values of θ and n.

$\lambda \downarrow$ $n \rightarrow$	20	40	60	80	100	200
0.1	0.769	0.850	0.898	0.929	0.950	0.990
	(0.704)	(0.817)	(0.882)	(0.921)	(0.949)	(0.989)
0.5	0.636	0.689	0.726	0.757	0.782	0.864
	(0.530)	(0.629)	(0.669)	(0.715)	(0.747)	(0.844)
0.9	0.587	0.622	0.648	0.670	0.688	0.756
	(0.478)	(0.538)	(0.582)	(0.62)	(0.643)	(0.724)
1.3	0.562	0.587	0.606	0.622	0.635	0.688
	(0.440)	(0.506)	(0.533)	(0.568)	(0.582)	(0.640)
1.5	0.553	0.575	0.591	0.605	0.617	0.664
	(0.431)	(0.491)	(0.528)	(0.544)	(0.564)	(0.623)
2.0	0.539	0.554	0.567	0.577	0.586	0.620
	(0.409)	(0.47)	(0.497)	(0.512)	(0.529)	(0.578)
2.5	0.529	0.542	0.551	0.559	0.566	0.592
	(0.408)	(0.448)	(0.472)	(0.496)	(0.510)	(0.552)

Table 8: PCS based on asymptotic and simulated (on parenthesis) results when the data come from $Exp(\lambda)$ distribution for different values of λ, n, and 10% of censored observations.

Figure 7: Screenshot of Tables 6, 7, and 8 from the main paper.

References

[1] Posada-Quintero, H. F., & Bolkhovsky, J. B. (2019). Machine Learning models for the Identification of Cognitive Tasks using Autonomic Reactions from Heart Rate Variability and Electrodermal Activity. *Behavioral Sciences*, **9(45)**. doi: 10.3390/bs9040045.
